# Senescence-secreted factors activate Myc and sensitize pretransformed cells to TRAIL-induced apoptosis

**DOI:** 10.1111/acel.12197

**Published:** 2014-03-04

**Authors:** Jelena Vjetrovic, Pattabhiraman Shankaranarayanan, Marco A Mendoza-Parra, Hinrich Gronemeyer

**Affiliations:** Department Functional Genomics and Cancer, Equipe Labellisée Ligue Contre le Cancer, Institut Génétique de Biologie Moléculaire et Cellulaire (IGBMC), CNRS/INSERM/UdS/CERBMBP 10142, 67404, Illkirch-Cedex, C.U. de Strasbourg, France

**Keywords:** apoptosis, Myc, secretome, senescence, TRAIL, tumor

## Abstract

Senescent cells secrete a plethora of factors with potent paracrine signaling capacity. Strikingly, senescence, which acts as defense against cell transformation, exerts pro-tumorigenic activities through its secretome by promoting tumor-specific features, such as cellular proliferation, epithelial-mesenchymal transition and invasiveness. Tumor necrosis factor-related apoptosis-inducing ligand (TRAIL) has the unique activity of activating cell death exclusively in tumor cells. Given that the senescence-associated secretome (SAS) supports cell transformation, we asked whether SAS factor(s) would establish a program required for the acquisition of TRAIL sensitivity. We found that conditioned media from several types of senescent cells (CMS) efficiently sensitized pretransformed cells to TRAIL, while the same was not observed with normal or immortalized cells. Dynamic transcription profiling of CMS-exposed pretransformed cells indicated a paracrine autoregulatory loop of SAS factors and a dominant role of CMS-induced MYC. Sensitization to TRAIL coincided with and depended on MYC upregulation and massive changes in gene regulation. Senescent cell-induced MYC silenced its target gene *CFLAR*, encoding the apoptosis inhibitor FLIP_L_, thus leading to the acquisition of TRAIL sensitivity. Altogether, our results reveal that senescent cell-secreted factors exert a TRAIL-sensitizing effect on pretransformed cells by modulating the expression of *MYC* and *CFLAR*. Notably, CMS dose-dependent sensitization to TRAIL was observed with TRAIL-insensitive cancer cells and confirmed in co-culture experiments. Dissection and characterization of TRAIL-sensitizing CMS factors and the associated signaling pathway(s) will not only provide a mechanistic insight into the acquisition of TRAIL sensitivity but may lead to novel concepts for apoptogenic therapies of premalignant and TRAIL-resistant tumors.

## Introduction

Normal proliferating cells undergo permanent growth arrest (senescence) in response to diverse environmental and endogenous stimuli such as telomere attrition, DNA damage, chemotherapy, oxidative stress, genotoxic chemicals, radiation, and oncogenes (Braig & Schmitt, 2006; Coppe *et al*., 2010). Senescent cells can even be generated from cancer cells during chemotherapy (Chang *et al*., 1999; te Poele *et al*., 2002). Importantly, both apoptosis and senescence within a cancer cell population contribute to the efficiency of anticancer therapies (Schmitt *et al*., 2002). Senescent cells exhibit a discernable phenotype with large and flattened morphology and exhibit an altered gene expression pattern, characterized by the senescence-associated secretome (SAS) (Coppe *et al*., 2008), also referred to as senescence-messaging secretome (SMS) (Kuilman & Peeper, 2009); SAS/SMS comprises of a variety of matrix metalloproteases, cytokines, and growth factors.

As many potentially oncogenic stimuli cause growth arrest in normal cells, senescence was considered a potent tumor-suppressive mechanism (Serrano *et al*., 1997; Campisi, 2001). However, it is now clear that senescent cells have also tumor-promoting potential, as they create a favorable microenvironment for tumor development by secreting specific SAS/SMS factors. Thus, senescent cells, which themselves cannot form tumors, promote the malignant transformation of adjacent premalignant cells (Campisi, 1997). Increasing evidence reveals moreover that senescent cells disrupt normal tissue structure and function (Parrinello *et al*., 2005), promote angiogenesis (Coppe *et al*., 2006), epithelial-mesenchymal transition and invasiveness (Coppe *et al*., 2008), and importantly, promote proliferation of premalignant and malignant cells *in vitro* (Krtolica *et al*., 2001; Liu & Hornsby, 2007), thus aiding tumor formation. Interestingly, secretory phenotypes similar to SAS have also been observed in fibroblasts adjacent to some carcinomas (Rodier *et al*., 2007), and senescent cells have been detected in both premalignant lesions (Campisi, 2005; Green, 2008) and around primary and secondary tumor sites (Charalambous *et al*., 2007). However, these studies were carried out with cancer cells for which the normal equivalent is not available, thus complicating the analysis of the molecular pathways that occur upon transformation of a normal cell to a tumor cell.

**Figure 1:**
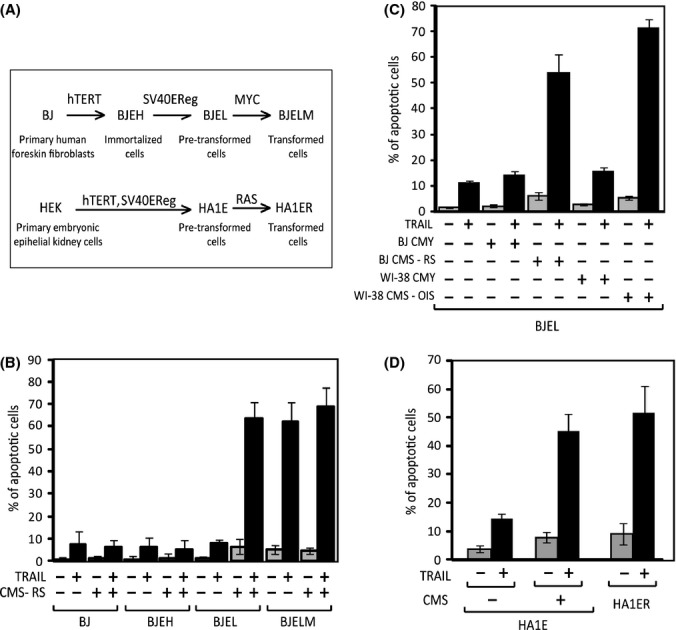
Conditioned medium from senescent cells (CMS) specifically sensitizes pretransformed cells to TRAIL-induced apoptosis. (A) The stepwise tumorigenesis model systems comprise (top) normal human foreskin fibroblasts (BJ), immortalized (BJEH), pretransformed (BJEL), and transformed (BJELM) cells, obtained through sequential overexpression of h-TERT, SV40-ER, and Myc ER, and (bottom) normal human embryonic epithelial kidney cells (HEK), pretransformed (HA1E), and H-*Ras*V12-transformed (HA1ER) cells. (B) TRAIL-induced apoptosis assays, cells of BJ system pretreated (+) or not (−) with conditioned medium from senescent cells (CMS) for 20 h, and then with 200 ng mL^−1^ of TRAIL for 24 h. Apoptosis measured by FACS analysis, using 7A6 (APO2.7) antibody (C) TRAIL-induced apoptosis of BJEL cells treated with two types of CMS (oncogene-induced senescence-OIS and replicative senescence-RS) and their nonsenescent counterparts. OIS cells obtained by stably overexpressing Ras in WI-38 fibroblasts (‘WI-38 CMS-OIS’), nontransfected WI-38 cells (‘WI-38 CMY’) were used as their nonsenescent equivalent. Replicative senescence cells were obtained through serial passaging of BJ cells (‘BJ CMS-RS’), young proliferating BJ fibroblasts used as their nonsenescent counterpart (‘BJ CMY’). (D) TRAIL-induced apoptosis in pretransformed and transformed cells of the HEK stepwise tumorigenesis system. Pretransformed HA1E cells were pretreated (+) or not (−) with conditioned medium from senescent cells (CMS) and then with TRAIL as in (B).

Despite the complexity of neoplastic transformation, model systems have been successfully developed in which primary human cells are transformed to tumor cells in a stepwise manner by the introduction of defined genetic elements (Hahn & Weinberg, 2002; Hanahan & Weinberg, 2011). In the present study, human foreskin BJ fibroblasts were first immortalized by the introduction of the telomerase catalytic subunit (BJEH cells). Subsequently, expression of SV40 early region (SV40EReg, expressing large T and small t) inactivated the pRb and p53 tumor suppressors and led to the formation of pretransformed BJEL cells. Complete transformation was achieved by overexpression of *MYC*, generating BJELM cells. This model (BJ-BJEH-BJEL-BJELM) system (Fig. 1A) provides a versatile system to study tumorigenesis in a virtually isogenic background. In addition, we used another stepwise system that involves normal (HEK), pretransformed (HA1E), and transformed (*Ras*V12 overexpressing, HA1ER) human embryonic kidney cells.

Tumor necrosis factor-related apoptosis-inducing ligand (TRAIL/Apo2L/TNFSF10) specifically targets fully transformed cells without affecting normal ones as confirmed in a variety of model systems (Ashkenazi & Eckhardt, 2008). Although its action is restricted to transformed cells, TRAIL does not kill all cancer cells equally efficient, as they may have developed resistance during the process of tumorigenesis or cancer progression. TRAIL binds as homotrimer to five receptors, the two death receptors DR4 (TRAIL-R1 or TNFRSF10A) and DR5 (TRAIL-R2 or TNFRSF10B), the decoy receptors DcR1 (TRAIL-R3 or TNFRSF10C) and DcR2 (TRAIL-R4 or TNFRSF10D), and OPG (Ashkenazi & Eckhardt, 2008). TRAIL binding induces DR oligomerization and results in the formation of the death-inducing signaling complex (DISC), which contains pro-caspase-8. Death-inducing signaling complex assembly confers autocatalytic activity onto pro-caspase-8 and generates active caspase-8, thereby initiating the caspase cascade. Tumor necrosis factor-related apoptosis-inducing ligand signaling can engage both the extrinsic and intrinsic death pathways, probably depending on the particular cell type and is, importantly, part of innate immune system (Takeda *et al*., 2002). Indeed, the crucial role of TRAIL in tumor surveillance has been confirmed by knockout experiments (Cretney *et al*., 2002) and the protection against chemically induced skin carcinogenesis of TRAIL-overexpressing transgenic mice (Kedinger *et al*., 2011).

Tumor necrosis factor-related apoptosis-inducing ligand signaling and apoptosis can be regulated by a number of modulators at different levels. Myc is a critical determinant of TRAIL apoptosis, and its overexpression is sufficient to confer sensitivity to DR5 agonists (Wang *et al*., 2004) (see also Fig. 1B). Other TRAIL modulators belong to the Bcl-2 family (Willis *et al*., 2005) or are members of the IAP family (Loeder *et al*., 2009). Additional control is exerted by the FLICE-like inhibitory protein (FLIP, the product of *CFLAR*), which is structurally similar to pro-caspase-8. They both contain a caspase-like domain and two death effector domains, but the long form of FLIP (FLIP_L_) lacks residues required for the autocatalytic activity of caspase-8. As a result, FLIP_L_ can bind to the DISC complex as competitor of pro-caspase-8, but no active p18 fragment is produced (Krueger *et al*., 2001), thus blocking downstream caspase processing.

To date, studies performed on the paracrine action of senescent cells have mostly focused on the tumor-promoting effect of the senescence secretome but did not address the possible effects on the TRAIL pathway. This is of significant importance, as the possible priming of TRAIL-induced cell death by senescence-secreted factors may provide mechanistic insight into the tumor cell selectivity of TRAIL and potentially lead to novel cancer therapeutic concepts. To explore whether SAS/SMS that was previously shown to confer a tumor phenotype on premalignant cells would suffice for the acquisition of TRAIL sensitivity by such pretransformed cells, we used the stepwise tumorigenesis model. Our results show that senescence-secreted factors specifically sensitize pretransformed cells to TRAIL-induced apoptosis and that sensitization is mediated through a biphasic Myc-dominated gene programming. These programs involve early activation of SAS, inflammatory and anti-apoptogenic factor-encoding genes followed by a late phase, where components and regulatory factors of DNA and chromatin are repressed, concomitantly with downregulation of *CFLAR* and increased expression of the TRAIL receptor *TNFRSF10B/DR5*. Our results suggest that dissection of the senescence secretome and characterization of its various factors will reveal not only the tumor-promoting activities of senescence cells but also uncover the signals that lead to the activation of the TRAIL pathway in premalignant cells. The knowledge derived from studies of TRAIL sensitization by SAS/SMS factor(s) may identify novel therapeutic paradigms for intervention at benign tumor stages.

## Results

### Conditioned medium from senescent cells sensitizes pretransformed cells to TRAIL-induced apoptosis

After validating the tumor specificity of TRAIL action in the stepwise human tumorigenesis model (Hahn *et al*., 1999), we incubated the cells at different stages of transformation with conditioned medium from senescent cells (CMS) for 20 h and treated them with TRAIL. Strikingly, we observed a significant increase in apoptosis in the pretransformed BJEL cells, while the death rate of the other cell types was unaffected (Fig. 1B). Notably, CMS sensitized pretransformed cells to TRAIL-induced apoptosis similarly as overexpression of Myc. Conditioned medium from different types of senescent cells [replicative (‘CMS-RS’), oncogene-induced (‘CMS-OIS’), etoposide (‘CMS Etop’), or H_2_O_2_ (‘CMS H_2_O_2_’)-induced senescence] sensitized BJEL cells with similar efficacy (Figs 1C and S1). The SAS/SMS action was specific, as its sensitization potential was a property of senescence secretome, not seen with the nonsenescent, proliferating counterparts (Fig. 1C), nor upon serum deprivation (data not shown). Similarly, pretransformed HA1E cells were also sensitized to TRAIL-induced apoptosis by SAS action (Figs 1D and S1). These results demonstrate that factor(s) specifically secreted by senescent cells endow pretransformed cells with TRAIL sensitivity and that this effect can be uncoupled from the senescence inducer.

### Analysis of the dynamic regulation of gene expression reveals distinct CMS-initiated MYC-dominated pathways and a paracrine regulatory loop of SAS expression

To understand the gene-regulatory effects of CMS, we monitored the gene expression changes in pretransformed BJEL cells at six time points during their sensitization to TRAIL. The temporal profiles of CMS-induced changes were analyzed by the Dynamic Regulatory Events Miner (Schulz *et al*., 2012) using the ChIP-X database, which associates 202 transcription factors with 46 988 target genes for TF-target gene annotations (Lachmann *et al*., 2010). Dynamic Regulatory Events Miner reconstructs a dynamic regulatory map by classifying genes into paths based on their co-expression patterns, predicts bifurcation points (BPs, time points where a novel co-expression pattern diverges from an ancestral one), and infers putative transcription factors (TFs) that are associated to a specific bifurcation event or a path.

**Figure 2:**
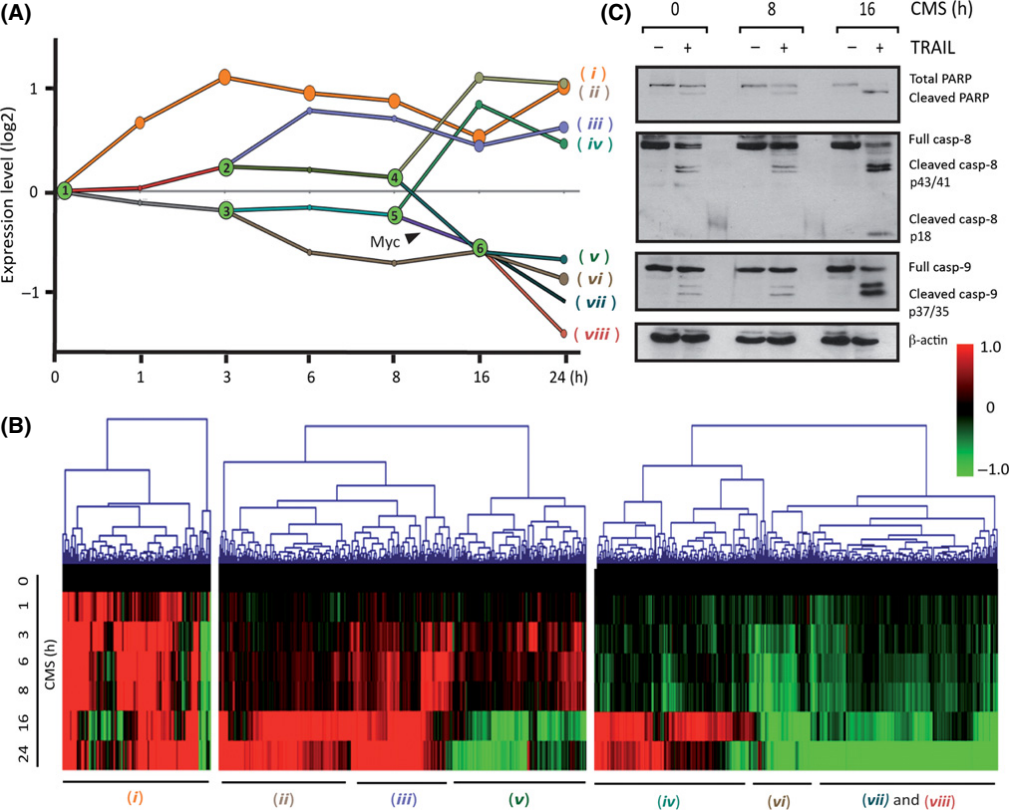
Dynamic regulatory map of CMS-induced sensitization to TRAIL sensitivity in BJEL cells and its analysis. (A) DREM generated dynamic regulatory map, with six bifurcation points (BPs) in green and paths stemming from them. Microarray gene expression data are represented in log2 values, Myc association to a specific path is noted. (B) Heatmap representing hierarchical clustering, Euclidean distance, complete linkage. (C) Western blot analysis of protein extracts from BJEL cells preincubated with CMS for 0, 8, or 16 h and treated with TRAIL. Immunoblots carried using apoptosis antibodies: anti-PARP, anticaspase-8, anticaspase-9, and actin antibody as a loading control.

The final DREM analysis generated 8 temporally staggered co-expression paths (*i* to *viii*) from six bifurcation points (Fig. 2A, BPs 1–6 in green). Hierarchical gene expression clustering for the components of the three separate paths generated by the first BP fully supported the DREM-based gene classification, as both methods produced highly similar cohorts (Fig. 2B).

While the expression of many genes was affected immediately, the largest overall divergence of global gene expression occurred 8 h after incubation with CMS (Fig. S2A). To define the point of commitment to TRAIL-induced apoptosis, we preincubated BJEL cells for 0, 8, or 16 h with CMS before treating them with TRAIL. Monitoring cleaved PARP, caspase-8, and caspase-9 revealed a clear acquisition of TRAIL sensitivity between 8 and 16 h (Fig. 2C), which correlated with massive gene expression changes. These data show that changes in gene expression that occurred at earlier time points are not sufficient for TRAIL sensitization and that gene regulation(s) critical for this phenomenon occur between 8 and 16 h.

The DREM analysis (Fig. 2A) revealed a set of genes with an immediate early and sustained positive response to CMS (*i*, in orange). GO term analysis and gene functional classification by DAVID indicated that this path was enriched for genes involved in negative regulation of apoptosis (e.g., *CFLAR, BCL2A1, BIRC3*) and inflammatory/immune, chemokine and growth responses, such as interleukins, chemokine ligands, or colony-stimulating factors (Table S1, Figs S2B and S3A). Unexpectedly, many of the heavily upregulated genes in path *i* (orange in Fig. 2A) encoded some of the same SAS factors that constitute the exogenously applied CMS. Indeed a comparison of the SAS components identified by antibody arrays (Coppe *et al*., 2008) (Fig. S2C, set A) with the gene sets upregulated at different time points during incubation of BJEL cells with CMS (sets B_1_, B_3_, B_24_) revealed that CMS induces expression of a large majority of SAS genes in BJEL cells, as validated by RT-qPCR for IL6, CCL2, and MMP3 (Fig. S2C, bottom panels). Importantly, the two main transcriptional regulators of SAS genes, NFκB and C/EBPβ were also upregulated by CMS (Fig. S2D, top panel). These data indicate that factors secreted from senescent cells induce a SAS-like transcriptome in pretransformed cells through a paracrine regulatory loop, which is in line with the observation that some SAS factors activate a self-amplifying secretory network thus reinforcing senescence and inducing proliferation arrest of adjacent normal cells (Acosta *et al*., 2008; Kuilman *et al*., 2008).

These immediate early gene-regulatory events are not only insufficient to convey TRAIL sensitivity to pretransformed BJEL cells but rather support resistance, as is suggested by the fact that genes encoding anti-apoptogenic factors, such as *CFLAR* and *BCL2A1,* are strongly induced within the first hours of CMS exposure and probably counteract the increased expression of the TRAIL death receptor *TNFRSF10B/DR5* (Fig. S2D, bottom panel).

**Figure 3:**
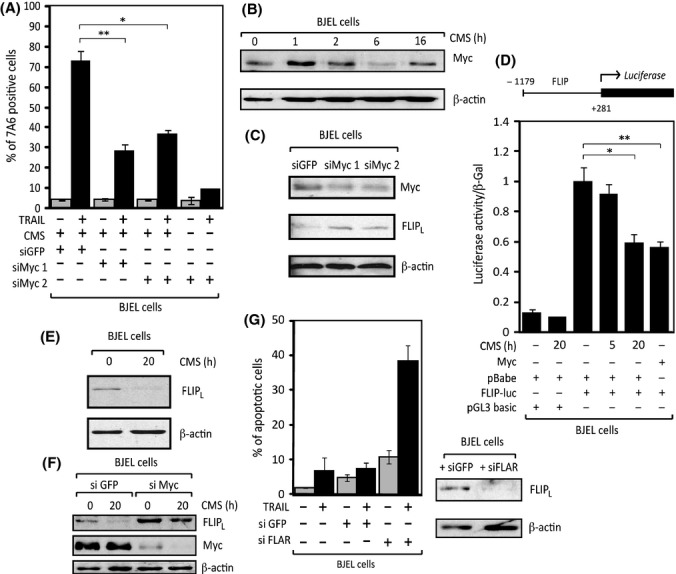
Myc and FLIP-L play a key role in sensitization to TRAIL-induced apoptosis by CMS. (A) Quantification of TRAIL-induced apoptosis in BJEL cells pretreated with siRNAs against Myc or siGFP as control for 24 h and then with CMS and TRAIL as usual. (B) Western blot analysis of total protein extracts from BJEL cells treated with CMS as specified using anti-Myc antibody and actin antibody as a loading control. (C) Western blot analysis of total protein extracts from BJEL cells treated with siRNAs against Myc or siGFP as a control for 24 h. Immunoblotting performed using anti-Myc antibody, anti-FLIP antibody, and actin antibody as a loading control. (D) Measurement of luciferase activity in BJEL cells transfected with FLIP-luc and Myc ER or FLIP-luc and pBabe. Cells were seeded in triplicates and treated with 4-OHT (Myc ER transfected cells, 10^−6^ m) or CMS for 5 and 20 h as indicated (pBabe-transfected cells). Luciferase activity, normalized to β-gal activity, is represented relative to pBabe at 0 h, which was given a value of 1. Statistically significant differences are shown (*P = 0.005 and **P < 0.0001). (E) Western blot analysis of total protein extracts from BJEL cells incubated with CMS for 20 h using anti-FLIP antibody and actin antibody as a loading control. (F) Western blot analysis of total protein extracts from BJEL cells transfected as in (C) and then treated with CMS for 20 h. All samples were collected together and immunoblotting was performed using anti-Myc antibody, anti-FLIP antibody, and actin antibody as a loading control. (G) TRAIL-induced apoptosis of BJEL cells transfected with siFLIP or siGFP as a control for 24 h and then treated with TRAIL as usual. (left panel). Western blot analysis of total protein extracts of siRNA-transfected BJEL cells at the moment of TRAIL treatment (right panel) using anti-FLIP antibody and actin as a loading control.

Given the apparent correlation between a major change of gene expression and acquisition of TRAIL sensitivity after 8 h exposure to CMS, we used DREM to identify the TF(s) responsible for the gene program(s) initiated at 8 h. Several TFs are associated to BP5 and BP6 and the paths emanating from these BPs. Intriguingly, Myc was the only transcription factor predicted with a high *P*-value [*P* = 0.002] to be involved in the decision underlying the acquisition of TRAIL sensitivity in the 8–16 h timeframe (Fig. 2A), and many of the repressed genes correspond to *bona fide* target genes of MYC (Fig. S3B). Thus, MYC is a key candidate to mediate CMS sensitization of pretransformed cells to TRAIL. This view is supported by the observation that the expression of *MYC* itself is regulated by CMS in these cells. Indeed, CMS induces both *MYC* RNA and protein levels generating a temporally staggered biphasic response (Figs 3B and S2D bottom panel), the first of which correlates with the induction of the immediate early genes in path *i*. It is worth noting that a great number of these genes are apparent MYC target genes, as is suggested by a comparison with MYC target sites inferred from ChIP-seq studies, which include MYC ChIP-seq profiling in the homologous BJ cell system in the context of re-programming experiments (Fig. S3A). However, as pointed out above, only the second peak of MYC expression and the regulation of a large number of MYC target genes (Figs S3B and C) coincide with the observed sensitization to TRAIL.

### Myc and FLIP_L_ are critical for sensitization to TRAIL-induced apoptosis by CMS

Overexpression of MYC is sufficient to sensitize pretransformed cells to TRAIL (Wang *et al*., 2005). Moreover, it is well established that MYC can act downstream of other oncogenes, such as H-*RAS*_V12_ (Sears *et al*., 2000). To explore whether CMS sensitization to TRAIL is mediated by MYC, we knocked it down using two different siRNAs and treated the BJEL cells 24 h later with CMS and TRAIL. Indeed, MYC knockdown, albeit incomplete, resulted in a significant reduction in CMS-induced TRAIL apoptosis compared to a siGFP control (Fig. 3A,C).

Interestingly, silencing of MYC increased the expression of anti-apoptogenic FLIP_L_ (Fig. 3C), in keeping with the observation that MYC can act as *CFLAR* repressor (Ricci *et al*., 2004). Note that expression of *CFLAR* (which encodes FLIP_L_) is stimulated by CMS, peaking at 3 h, and becomes heavily downregulated during the second wave of *MYC* induction and the acquisition of TRAIL sensitivity (Fig. S2D bottom panel), indicating that MYC activity causes the silencing of FLIP_L_. Luciferase reporter assays further supported that FLIP is repressed by MYC, as *MYC* overexpression inhibited the activity of chimeric *FLIP-Luciferase* reporter in BJEL cells (Fig. 3D). Importantly, incubation of these cells with CMS for 20 h produced the same repression. Moreover, FLIP_L_ protein levels decreased in CMS-incubated BJEL cells after the same incubation time (Fig. 3E). Finally, when siMyc-treated BJEL cells were additionally incubated with CMS, we observed not only an expected higher initial amount of FLIP, but also a failure of CMS to downregulate FLIP_L_ levels under conditions of MYC depletion (Fig. 3F). In keeping with the observation that *CFLAR* expression (Fig. S2D bottom panel) and FLIP_L_ protein levels (Fig. 3E) declined at the time of acquisition of TRAIL sensitivity, we concluded that FLIP_L_ repression by CMS is controlled by MYC. FLIP_L_ was indeed the factor that enables TRAIL to induce apoptosis, as siRNA-based FLIP_L_ knockdown was sufficient to sensitize pretransformed (BJEL) cells to TRAIL-induced apoptosis (Fig. 3G). Taken together, these results show that conditioned medium from senescent cells decreases FLIP_L_ levels via MYC-mediated repression and confers sensitivity to otherwise TRAIL-resistant pretransformed cells.

**Figure 4:**
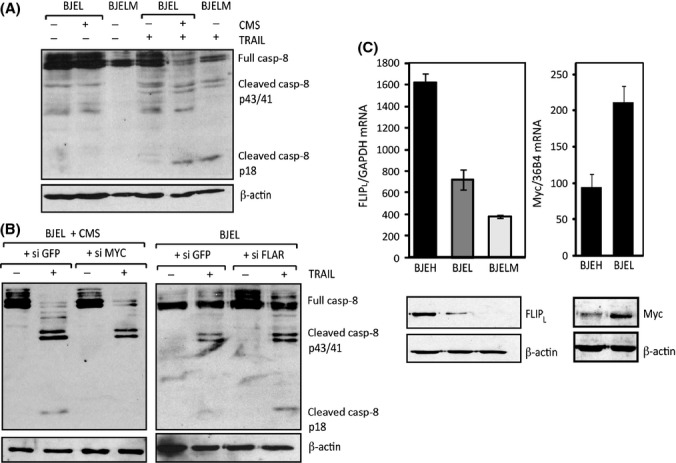
FLIP-L inhibits sensitivity of cells to TRAIL by blocking full cleavage of caspase-8 and formation of p-18 fragment. (A) Western blot analysis of total protein extracts from BJEL and BJELM cells treated with CMS as indicated and then with TRAIL for 3 h. (B) BJEL cells were pretreated with siMyc and siGFP as a control (left panel) or siFLAR and siGFP as a control and then treated with CMS as in A (right panel). Immunoblots were performed using anticaspase 8 antibody and actin antibody as a loading control. (C) Comparison of FLIP_L_ and MYC RNA and protein levels. Reverse transcription–PCR was performed on total RNA extracted from cells of the BJ system. *CFLAR* mRNA was normalized to GAPDH (upper left), *MYC* RNA to 36B4 (upper right). Western blot analysis of total protein extracts was performed with anti-FLIP_L_ or anti-MYC antibodies using actin as loading control (lower panel).

The long form of FLIP prevents efficient cleavage of pro-caspase-8 resulting in inefficient apoptosis induction (Krueger *et al*., 2001). To determine the step(s) crucial for the acquisition of TRAIL sensitivity in BJEL cells, we compared initiator pro-caspase-8 activity of BJEL, CMS-treated BJEL cells, and fully transformed cells that go readily into apoptosis upon TRAIL treatment. No p18-cleaved caspase-8 was observed in TRAIL-treated BJEL cells, whereas incubation of BJEL cells with CMS resulted in complete pro-caspase-8 cleavage and formation of active p18, like in BJELM cells (Fig. 4A). The absence of active caspase-8 reflects a pre-existing insensitivity of BJEL cells to TRAIL necessitating decreased FLIP levels to commit to apoptosis. Inversely, BJEL cells treated with siMyc prior to treatment with CMS and TRAIL showed incomplete caspase-8 cleavage and did not generate active p18 (Fig. 4B, left panel). Thus, knockdown of Myc prevents CMS sensitization to TRAIL by blocking the decrease in FLIP_L_ levels, which ultimately inhibits generation of active p18 caspase-8. In support of this model, siRNA-mediated FLIP knockdown was sufficient to ensure full cleavage of pro-caspase-8 (Fig. 4B, right panel).

To understand the observed specificity of CMS action for pretransformed cells in the stepwise tumorigenesis model, we monitored *CFLAR* expression. Interestingly, we observed a progressive decrease in *CFLAR* mRNA and FLIP_L_ protein levels toward the transformed cells (Fig. 4C, left panels), which was inversely correlated with *Myc* expression (Fig. 4C, right panels). We concluded that during the transformation process, SV40EReg provides a cellular and molecular context that synergizes with the senescent secretory profile to promote sensitization toward TRAIL. Together, these observations suggest that relative to TRAIL-resistant BJ or BJEH cells, FLIP_L_ levels in BJEL cells decreased due to the introduction of SV40EReg and that CMS incubation further silences *CFLAR* expression to levels compatible with TRAIL-induced apoptosis.

### CMS sensitization of cancer cells

**Figure 5:**
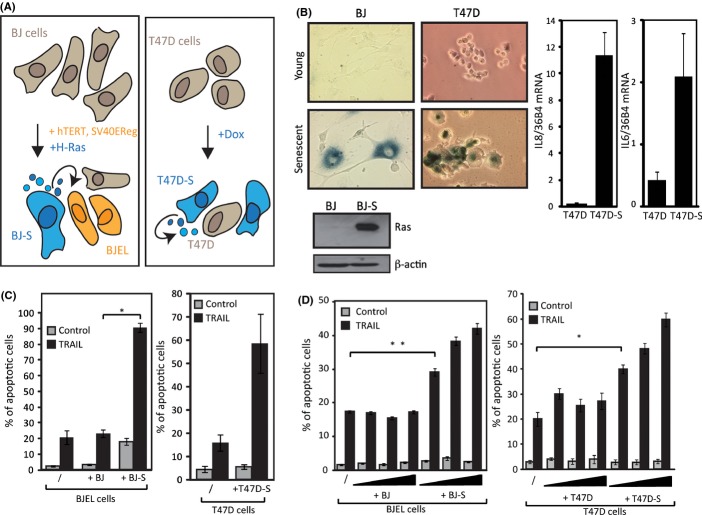
Sensitization to TRAIL in cancer cells and in co-culture conditions. (A) Generation of senescent and pretransformed cells from BJ cells, through genetic changes. Pretransformed (BJEL) cell, obtained by Hahn and Weinberg after introduction of hTERT and SV40EReg and senescent (BJ-S) cells are formed through H-Ras overexpression in BJ cells. Senescent (T47D-S) cells were obtained after doxorubicin treatment of TRAIL-unresponsive T47D cancer cells. (B) Comparison of young and senescent cells. Senescence-associated β-gal (SA β-Gal) staining of young (BJ, T47D) and their derivative senescent (BJ-S, T47D-S) cells (20×, upper left panel). Immunoblots with protein extracts from BJ and BJ-S cells with anti-H-Ras and actin antibodies (lower left panel). Reverse transcription–PCR was performed on RNA extracted from T47D and T47D-S cells. IL6 and IL8 mRNA were normalized to 36B4 (right panel). (C) Quantification of TRAIL-induced apoptosis in BJEL or T47D cells grown alone (/) or incubated with conditioned medium from appropriate young (BJ, T47D) or senescent (BJ-S, T47D-S) cell. (D) Co-culture of BJEL or T47D cells with BJ/T47D or BJ-S/T47D-S cells at increasing confluences, mean, and SEM of triplicates;**P < 0.005 and *P < 0.03.

The above results demonstrate that factors secreted from senescent cells sensitize pretransformed cells to TRAIL. We wondered whether the same phenomenon would occur when senescent and pretransformed cells were derived from the same origin (illustrated in Fig. 5A). To address this, pretransformed BJEL cells were exposed to CMS from H-*RAS*_V12_ OIS-induced senescent (BJ-S) cells that displayed typical senescent cells characteristics (Fig. 5B, left panels). We observed a clear sensitization to TRAIL, while there was no sensitization with medium from young cells (Fig. 5C, left panel). Pretransformed cells were then co-incubated with increasing amounts of either senescent or young/replicating cells, and while young cells (BJ) had no effect on the TRAIL sensitivity of BJEL cells, there was a clear dose-dependent sensitization by senescent cells (Fig. 5D, left panel). Thus, OIS-induced senescent cells can sensitize pretransformed cells of the same origin to TRAIL.

The observations that tumor cells can acquire a senescent phenotype upon chemotherapeutic treatment (Chang *et al*., 1999; te Poele *et al*., 2002) prompted us to explore whether TRAIL-resistant cancer cells that are induced to undergo senescence and secrete SAS/SMS factors would confer TRAIL sensitivity to adjacent sister cells. For this, we used the human ductal breast epithelial tumor T47D cells, which show only residual response toward TRAIL (Fig. 5C, right panel) and doxorubicin-generated senescent T47D cells (T47D-S, illustrated in Fig. 5A) (Rastogi *et al*., 2006). These cells display the expected characteristics of senescent cells; they were growth-arrested and increased in size, stained positively for β-galactosidase, and expressed increased levels of IL6 and IL8 (Fig. 5B, right panels). Notably, the CMS collected from these cells sensitized unresponsive T47D cells to TRAIL (Fig. 5C, right panel). Moreover, in co-culture experiments, T47D cells responded to TRAIL treatment in a T47D-S dose-dependent manner (Fig. 5D, right panel). We thus demonstrated sensitization to TRAIL within a population of tumor cells, if a fraction of those cells commits to therapy-induced senescence. The fact that senescent cells have been found in cancer tissue after chemotherapeutic treatments of patients (te Poele *et al*., 2002) underscores the potential therapeutic implication of this observation.

## Discussion

We have demonstrated that factors secreted from senescent cells sensitize ‘pretransformed’ cells, in which p53 and Rb actions are impaired, to TRAIL. This sensitization was seen with conditioned medium from various types of senescent cells that had entered replicative, oncogene, or chemotherapy-induced senescence. Using the BJ stepwise human cellular tumorigenesis model, we show that this process is highly selective, as (i) only senescent cell medium had the ability to sensitize to TRAIL and (ii) only pretransformed cells could be sensitized, while normal (BJ) or immortalized (BJEH) cells remained unresponsive. We conclude from these data that only cells that have already undergone initial steps toward malignancy are sensitized to TRAIL by senescence-secreted factors. That siRNA-mediated p53 knockdown did not result in TRAIL sensitization of BJEH cells by CMS, as seen in BJEL cells (Fig. S4), suggests that additional impairment of Rb and/or other signaling pathways are required for sensitization.

Several studies have explored the composition and the nature of the senescence secretome in great detail (Kuilman & Peeper, 2009; Coppe *et al*., 2010). As IL6 has been identified as one of the key factors involved in paracrine mechanisms governed by senescent cells (Coppe *et al*., 2008; Kuilman *et al*., 2008), we tested early on whether IL6 could be the factor that induces the acquisition of TRAIL sensitivity. However, our data reveal that on its own, IL6 is not sufficient to sensitize BJEL cells. Although it is highly upregulated and secreted by senescent cells, CMS incubation does not lead to significant IL6-induced phosphorylation of Stat3 in BJEL cells (Fig. S5A), compared to HepG2 cells, frequently used to test IL6 and IL6-neutralizing antibody action (Kuilman *et al*., 2008). Additionally, we detected rather low levels of *IL6 receptor* mRNA in BJEL cells compared to HepG2 ones (Fig. S5B). Similarly, IL6-neutralizing antibody failed to block CMS-induced sensitization of BJEL cells to TRAIL (Fig. S5C). It is thus likely that other SAS factors or a cocktail of them, possibly together with gene-regulatory events (Table S1) induced by the senescence secretome, generate a cellular milieu that is compatible with the apoptogenic action of TRAIL. In this respect, our DREM analysis provided detailed insight into the dynamic gene regulation initiated by the CMS secretome in a paracrine fashion. Our attempts to identify the TRAIL-sensitizing SAS component(s) revealed unexpected features, as the activity was found very stable to denaturation conditions and proteolytic enzymes and was found in a fraction of < 3 kDa (Fig. S6). However, up to now, we have not been able to attribute this activity to an individual compound or HPLC signature.

Importantly, we identified two major temporally staggered waves of gene regulation. The immediate early wave comprised as important components anti-apoptosis signaling, which possibly supports temporally the TRAIL-resistant phenotype and an unexpected SAS component suggestive of a paracrine regulatory loop reminiscent of similar phenomena observed in other systems (Acosta *et al*., 2008; Kuilman *et al*., 2008). However, the most dramatic changes in gene expression occurred between 8 and 16 h when pretransformed cells acquire TRAIL sensitivity. DREM predicted major MYC-dependent negative gene regulation to occur during this time, and indeed, many of the repressed genes, including the anti-apoptogenic *CFLAR*, have been classified as MYC targets. Notably, CMS induces a biphasic expression of MYC resulting in an early and a late peak. The early peak correlates with predominantly positive gene regulation and multiple of those genes are putative MYC targets (Fig. S3A). We speculate that differential heterodimer formation and/or post-translational modifications may account for the divergent regulation of MYC targets at the early and late phases.

Concentrating on the second peak of *MYC* expression, we confirmed that MYC is indeed required for TRAIL sensitization of pretransformed cells. Our mechanistic studies show that MYC-dependent repression of FLIP_L,_ an anti-apoptotic factor that inhibits TRAIL action at the level of the death-inducing signaling complex and an established negatively regulated target gene of *MYC* (Ricci *et al*., 2004), is key in the acquisition of TRAIL sensitivity by CMS-exposed BJEL cells.

It is important to point out the *MYC* expression is frequently deregulated in cancer cells; indeed, its expression is increased in a wide variety of human cancers, including 80% of breast cancer, 70% of colon cancer, and 50% of hepatocellular carcinomas (Gardner *et al*., 2002). This suggests that a large number of human primary cancers may, as far as the MYC-FLIP_L_ regulon is concerned, have the potential to respond to TRAIL.

Sensitizing cells to TRAIL by reducing FLIP_L_ levels is a widely recognized mechanism (Geserick *et al*., 2008; Zhang *et al*., 2010) and was shown to be involved in tumor regression (Ganapathy *et al*., 2009) Intriguingly, increased FLIP_L_ levels were observed during transformation and melanoma formation as a mechanism of developing intrinsic resistance to apoptosis (Rippo *et al*., 2004). Thus, the observation that senescent cells can affect FLIP_L_ levels identifies a paracrine mechanism that could be potentially utilized for modulating resistance through induction of senescence (see below).

Our data reveal a dichotomy of pro- and antitumorigenic action of senescent cells and their secretome. On the one hand, CMS confers TRAIL sensitivity by direct signaling to pretransformed cells, which are at an early stage in the process of tumorigenesis and represent a highly important target for therapy. On the other hand, previous results have established a clear tumor-promoting effect of senescent cells (Krtolica *et al*., 2001). Indeed, we have recapitulated these effects with the stepwise transformation system (Fig. S7). Therefore, it will be important to dissect the pro- and antitumorigenic activities of the senescence secretome to pave the way toward a novel type of TRAIL-based apoptogenic therapy that targets premalignant/benign cancers.

Importantly, TRAIL sensitization by senescent cells and their secretome is not limited to premalignant cells. Indeed, we also sensitized established tumor cells when senescence was induced by chemotherapy within a population of same cancer cells. Moreover, chemotherapeutic treatment of TRAIL-insensitive tumor cells (T47D) induced senescence and sensitized adjacent (co-cultured) resistant tumor cells to TRAIL-induced apoptosis. While the corresponding mechanisms are incompletely understood, the ability of chemotherapeutic drugs to induce senescence in tumors is well established (reviewed by Shay & Roninson, 2004). In conclusion, the senescence secretome may sensitize both premalignant cells and certain TRAIL-resistant tumors upon chemotherapy, thus providing a rationale for the co-administration of TRAIL or TRAIL pathway-activating therapeutics.

Targeted destruction of cancer cells with minimal damage to normal cells is the ultimate aim of cancer therapy (for a recent review see Pavet *et al*., 2011). Multiple TRAIL-based (combo) therapies are currently explored *in vitro* and in clinical trials, including the cancer therapeutic potential of recombinant TRAIL, humanized TRAIL receptor-activating antibodies, TRAIL mimetic peptides, and combinatorial treatments using recombinant TRAIL and irradiation or chemotherapeutics. Our findings that senescent cells can sensitize not yet fully transformed cells or unresponsive tumor cell lines to TRAIL provides a novel view on the interactomes of aging and cancer cells and may pave the way to novel therapeutic approaches.

## Experimental procedures

### Cell culture and reagents

Primary human diploid BJ foreskin fibroblasts were obtained from the American Type Culture Collection (ATCC). Genetically defined cells of BJ stepwise system (BJ, BJEH, and BJEL) and HEK stepwise system (HA1E – pretransformed and HA1ER-transformed cells) were generously provided by Drs. *Hahn* and *Weinberg*.

Oncogene-induced senescent (OIS) cells were obtained by oncogene (H-*RAS*_G12V_) overexpression in WI-38 (WI-38 Ras) and BJ cells (BJ-S). Replicative senescent BJ cells were obtained by continuous passaging, while T47D (T47D-S), H_2_O_2,_ and etoposide-induced senescence was obtained by the corresponding brief treatment (Data S1). Senescent cells were positive for several senescence-specific marks: increased size, growth arrest, senescence-associated β-Galactosidase staining, presence of SAHFs and their co-localization with H3K9me3 (data not shown), and increased RNA levels of senescence-specific factors. For routine technologies (cell and tissue staining, immunofluorescence, immunohistochemistry, Western blotting, transient transfections, reporter assays, xenografting), see Data S1 (Supporting information).

### Retroviral production and infection

BJELM cells were produced by retroviral transfection of BJEL cell with pBabe-Myc-ER and were continuously grown in medium with 10^−6^ m 4-hydroxytamoxyfen (4-OHT). Oncogene-induced senescent cells (WI-38 Ras and BJ-S cells) were produced by retroviral transfection with pBabe-RasG12V. Several days after transfection, cells ceased to grow and showed characteristic senescent profile. For retrovirus production, see Data S1 (Supporting information).

### Apoptosis measurement

Cells were seeded in 24-well plates (30 000 cell per well) and incubated (+) or not (−) with 1 mL of appropriate conditioned medium. Conditioned medium was collected after filtration (0.20-μm Minisart High-Flow syringe filter; Sartorius, Goettingen, Germany) of 2-day-old medium from senescent cells or replicating counterparts. After 20 h, cells were incubated with rhTRAIL (200 ng mL^−1^) for additional 16 h to monitor and measure apoptosis. Whole well content, with floating (apoptotic) and attached cells, was collected for apoptosis measurement. Cell pellets were permeabilized on ice with 100 μg mL^−1^ digitonin and stained with APO2.7 (1:5) (Beckman Coulter, Miami, FL, USA). Apoptosis was measured by FACS and quantified by detection of 7A6 mitochondrial antigen.

### RNA interference

Cells were seeded in 24-well (20 000 cells per well) or six-well plates (100 000 cells per well). Transfections were performed with 20 pmol per well (24-well plates) or 100 pmol per well (six-well plates) of appropriate siRNA. Transfections were performed using Lipofectamine 2000 (Invitrogen-Life Technologies, Saint Aubin, France) according to manufacturer’s protocol. Small interfering RNAs (siRNAs) used were as follows: siGFP (Amaxa, maxGFP, part of VSC-1001 siRNA test Kit for Cell Lines and Adherent Primary Cells), siGlo Transfection Indicators (Thermo Scientific Dharmacon-Fisher Scientific, Illkirch, France, D-001630-01). For Myc siRNA experiments, we used siMyc 1 – siGENOME SMARTpool Human Myc (Dharmacon) or siMyc 2 with the sense sequence 5′-GGUCAGAGUCUGGAUCACCTT-3′. For FLIP siRNA experiments, we used siGENOME SMARTpool siRNA HumanCFLAR (Dharmacon).

### RT-qPCR

Total RNA extracts were obtained using RNeasy Mini Kit (Qiagen, Hilden, Germany); cDNA was obtained using Transcriptor Reverse Transcriptase Kit (Roche Diagnostics, Meylan, France) following manufacturer’s instructions. PCR was performed using SYBR Green JumpStart Taq ReadyMix (S4438; Sigma, Lyon, France) and desired primers at 0.5 μm final concentration. The primer sequences used are specified in Table S2 (Supporting information).

### Microarrays

Pretransformed BJEL cells were incubated with CMS for 0, 1, 3, 6, 8, 16, or 24 h, and 1 μg of corresponding total RNA (100 ng mL^−1^) was hybridized on Affymetrix Human Gene 1.0 ST Arrays. Samples and GeneChips were processed following Affymetrix recommendations (Affymetrix, Santa Clara, CA, USA), and after chip scanning, raw image file (.DAT) was generated by Affymetrix Command Console (agcc v3.2). After alignment of a grid (.GRD file), a smoothened image file (.CEL) was created and used to calculate the signal intensity for each probeset on the chip using RMA algorithms with the Affymetrix Software Expression Console v 1.1 (default parameters: Sketch quantile normalization). The complete microarray data have been deposited at GEO (accession number GSE49944). The dataset can be accessed via the following link: http://www.ncbi.nlm.nih.gov/geo/query/acc.cgi?token=rxktjgmkysekyns&acc=GSE49944

### Dynamic Regulatory Events Miner analysis and other bioinformatic tools

We generated dynamic regulatory map with Dynamic Regulatory Events Miner (drem v2.0) (Ernst *et al*., 2007; Schulz *et al*., 2012). The time point transcriptomics data were expressed in log2 ratios relative to 0 h and were imported into DREM together with the ‘Transcription factor/genes they regulate’ input file (Lachmann *et al*., 2010). This unbiased approach led to establishment of co-expression paths, prediction of bifurcation points (BPs), and association of given transcription factors to a BP or a path, as estimated by enrichment of its target genes using hypergeometric distribution relative to the genes associated with a given BP or the common path.

To confirm the paths predicted by DREM, we used TMEV (Multi Experiment Viewer v4.8) and performed hierarchical clustering (Euclidean distance, complete linkage) of the genes from each of the three separate subsets stemming from the first BP that later form path *(i)* (first set), go through BP2 (second set) or BP3 (third set).

Venn diagrams were generated by comparing data obtained in our microarray at various time points with the antibody array results obtained by comparison of BJ sen/BJ pre 20% O_2_ (Coppe *et al*., 2008).

### Co-culture experiments

Thirty thousand of BJEL or T47D cells were seeded in the 24-well plates (Corning - Falcon, Hazebrouck, France, #353504), with cell culture inserts (Corning - Falcon, #353095, 0.4 μm) placed in the upper portion of the well and the appropriate number of cells seeded. For co-culture experiments, normal (BJ or T47D) or senescent (BJ-S or T47D-S) cells were seeded at increasing amounts (2.5, 5, or 10 × 10^3^ for BJ and 5, 10, or 20 × 10^3^ for BJ-S) in the upper inserts. Cells were grown for 3 days after which TRAIL was added (200 ng mL^−1^) and apoptosis was measured.

## Supporting Information

**Fig. S1** CMS from etoposide and H_2_O_2_-treated cells sensitizes pretransformed cells to TRAIL-induced apoptosis.

**Fig. S2** Specific features of DREM profiles.

**Fig. S3** Differential expression levels for genes retrieved in MYC-associated co-expression paths.

**Fig. S4** Knockdown of p53 on its own is not sufficient for sensitization of BJEH cells to TRAIL.

**Fig. S5**IL6 is on its own not sufficient for sensitization of BJEL cells to TRAIL.

**Fig. S6** Properties of TRAIL-sensitizing CMS compound(s).

**Fig. S7** Senescent cells enable pretransformed cells to form tumors in nude mice.

**Table S1**GO terms associated with paths defined by DREM analysis.

**Table S2** Primers used in quantitative PCR.

**Data S1** Experimental procedures.
