# Supplementary figures and images for: Senescence-secreted factors activate Myc and sensitize pretransformed cells to TRAIL-induced apoptosis
Source: Aging Cell. 2014 Mar 4;13(3):487–96. doi: 10.1111/acel.12197 (PMC4326894; doi:10.1111/acel.12197)

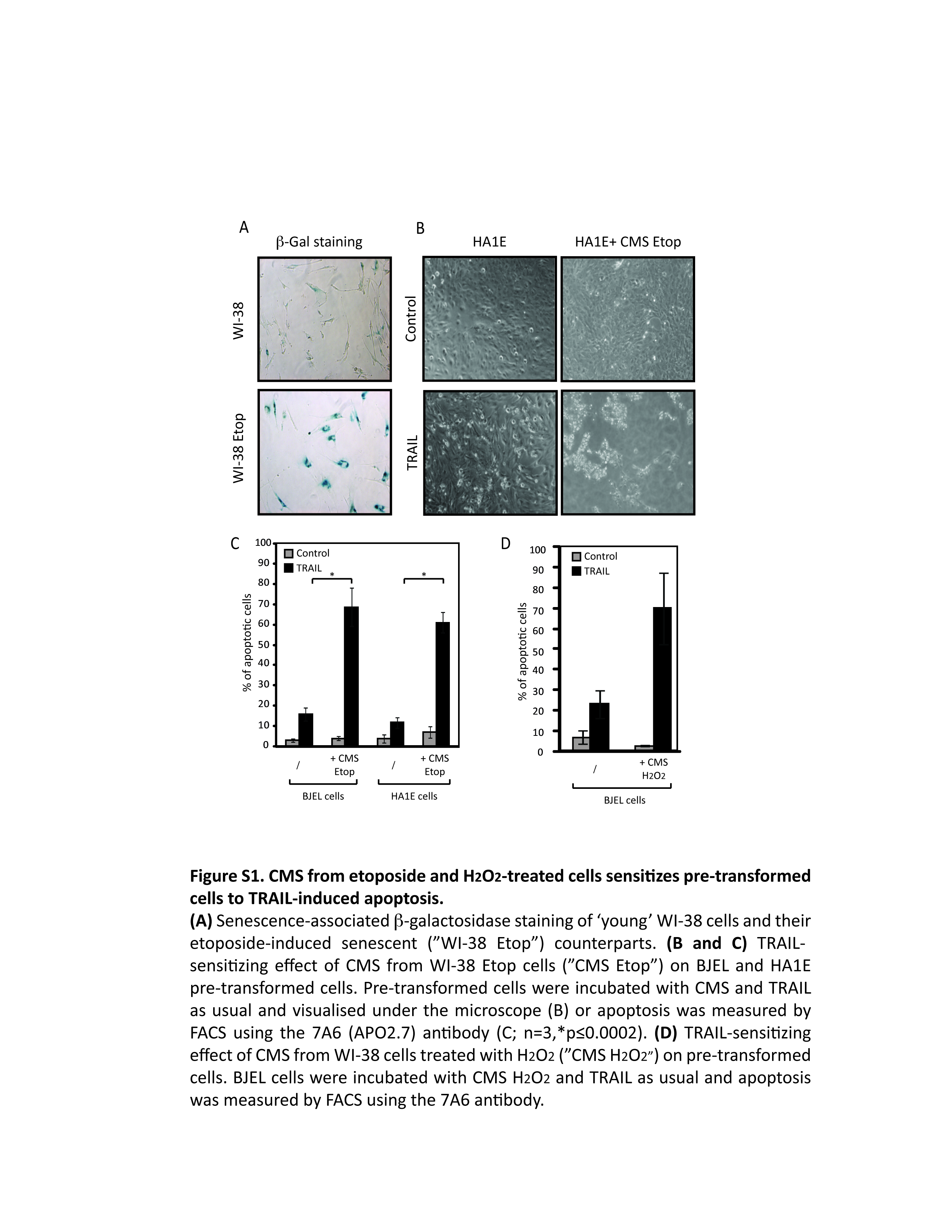

Supplement: Supplementary file 1 — Fig. S1 CMS from etoposide and H2O2-treated cells sensitizes pretransformed cells to TRAIL-induced apoptosis. [file acel0013-0487-sd1.tif]

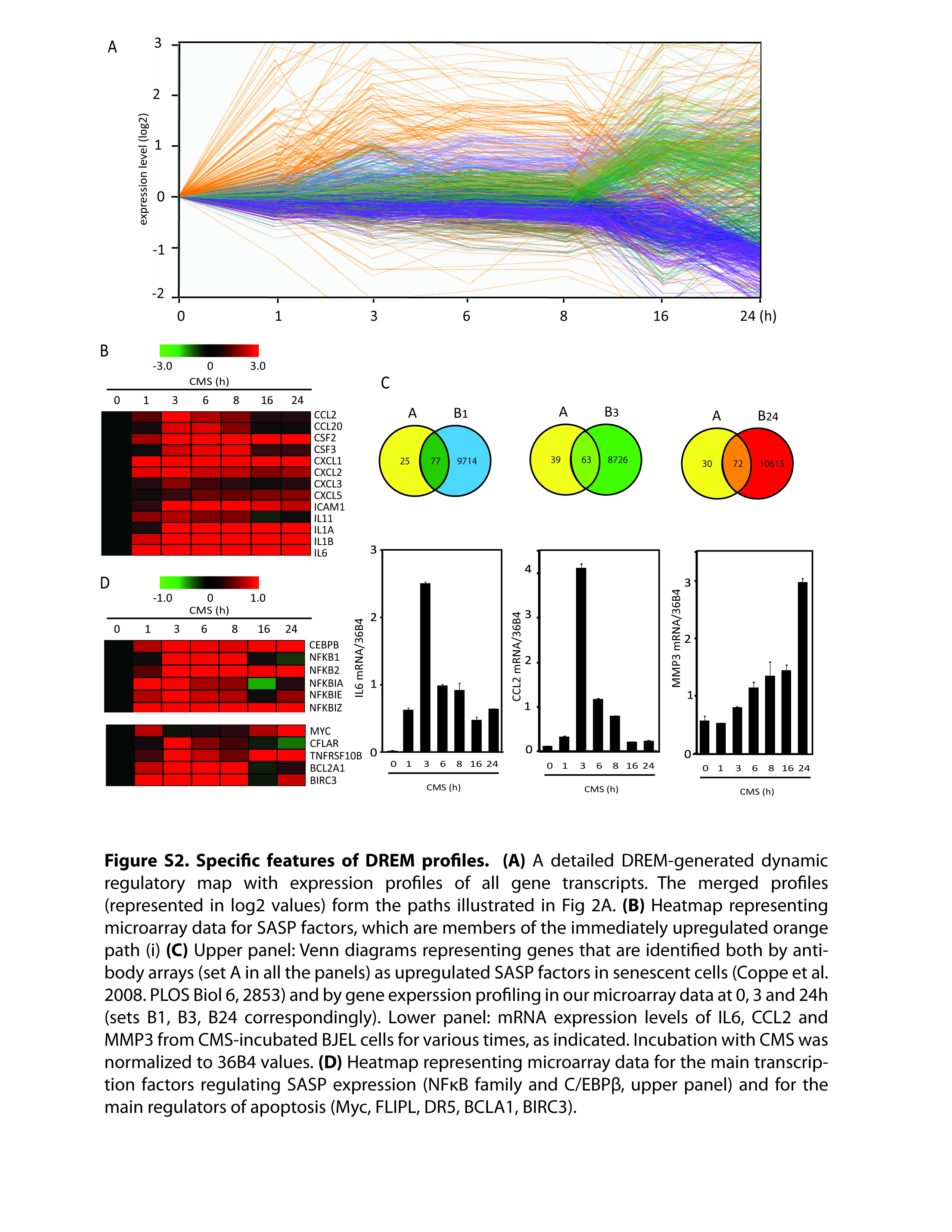

Supplement: Supplementary file 2 — Fig. S2 Specific features of DREM profiles. [file acel0013-0487-sd2.tif]

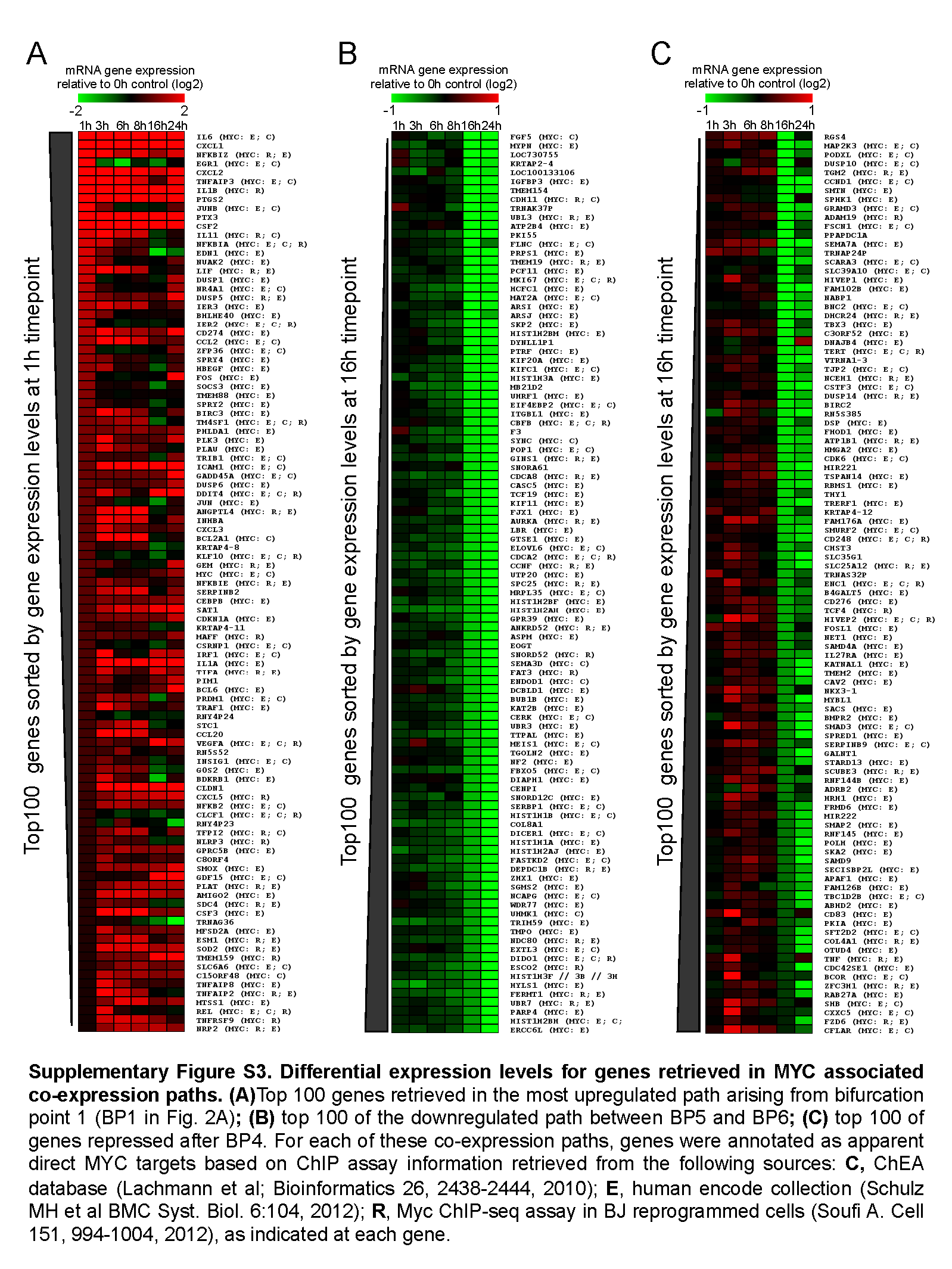

Supplement: Supplementary file 3 — Fig. S3 Differential expression levels for genes retrieved in MYC-associated co-expression paths. [file acel0013-0487-sd3.tiff]
