# Supplementary material for: Senescence-secreted factors activate Myc and sensitize pretransformed cells to TRAIL-induced apoptosis
Source: Aging Cell. 2014 Mar 4;13(3):487–96. doi: 10.1111/acel.12197 (PMC4326894; doi:10.1111/acel.12197)
Supplement: Supplementary file 4 — Fig. S4 Knockdown of p53 on its own is not sufficient for sensitization of BJEH cells to TRAIL. [file acel0013-0487-sd4.pdf]

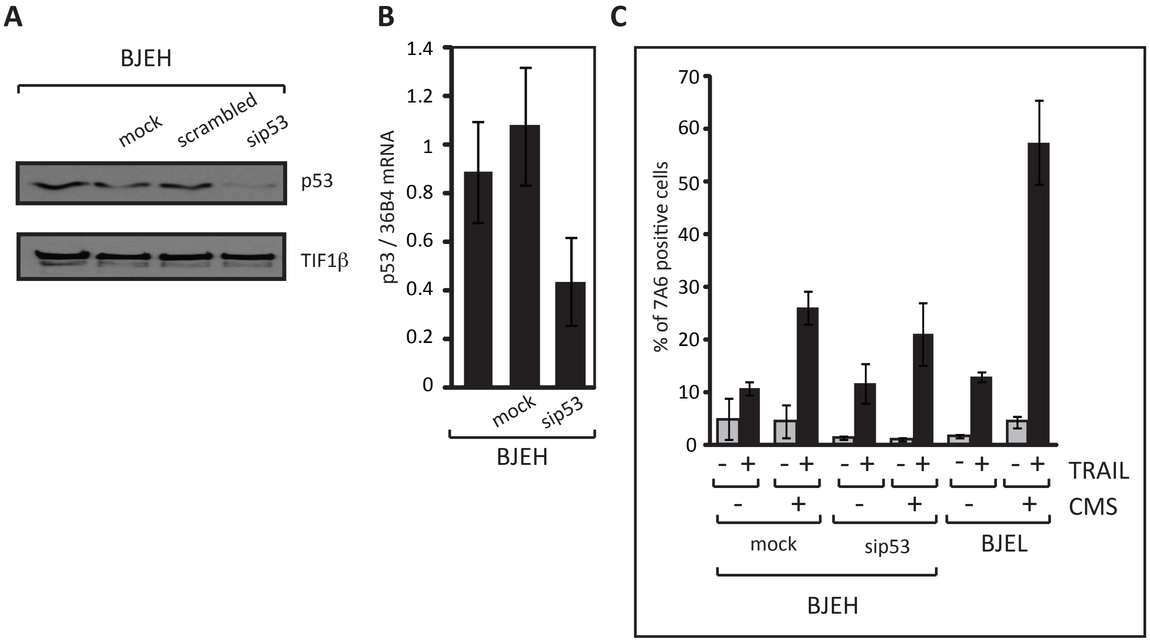

Figure S4. Knock down of p53 on its own is not sufficient for sensitization of BJEH cells to TRAIL.

(A) Western blot and **(B)** RT-PCR quantitation revealing siRNA-mediated knock down of p53 in BJEH cells. **(C)** CMS exposure of both mock and sip53 transfected BJEH cells generates only a weak TRAIL response (possibly related to transfection-induced stress) but does not lead to the sensitization seen inBJEL cells.
