# Supplementary material for: Senescence-secreted factors activate Myc and sensitize pretransformed cells to TRAIL-induced apoptosis
Source: Aging Cell. 2014 Mar 4;13(3):487–96. doi: 10.1111/acel.12197 (PMC4326894; doi:10.1111/acel.12197)
Supplement: Supplementary file 5 — Fig. S5IL6 is on its own not sufficient for sensitization of BJEL cells to TRAIL. [file acel0013-0487-sd5.pdf]

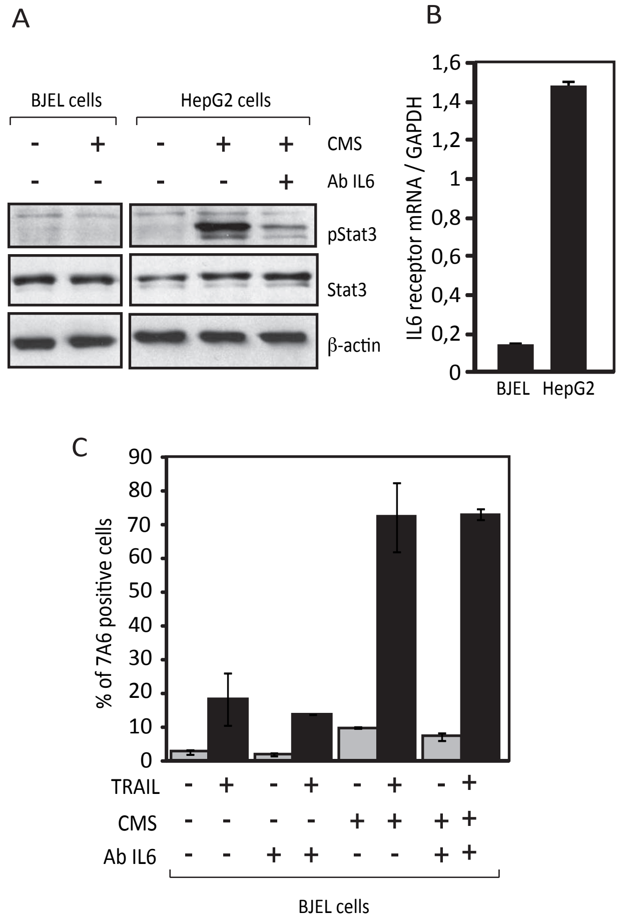

Figure S5. IL6 is on its own not sufficient for sensitization of BJEL cells to TRAIL.

(A) Serum-starved BJEL and HepG2 cells were stimulated for 15 minutes with CMS. As indicated some HepG2 cells were incubated for 30 min with 10 µM IL6-neutralizing antibody. Total protein extracts were collected and immunoblots were done using anti-phospho Stat3 antibody, anti-total Stat3 antibody and β-actin. **(B)** RNA was extracted from BJEL and HepG2 cells and after reverse-transcription PCR was performed using primers specific for *IL6R*. *IL6R* mRNA was normalized to *GAPDH* values. **(C)** Quantification of TRAIL-induced apoptosis in BJEL cells after treatment with 10 µM IL6-neutralizing antibody, CMS and/or TRAIL, as indicated.
