# Supplementary material for: Senescence-secreted factors activate Myc and sensitize pretransformed cells to TRAIL-induced apoptosis
Source: Aging Cell. 2014 Mar 4;13(3):487–96. doi: 10.1111/acel.12197 (PMC4326894; doi:10.1111/acel.12197)
Supplement: Supplementary file 6 — Fig. S6 Properties of TRAIL-sensitizing CMS compound(s). [file acel0013-0487-sd6.pdf]

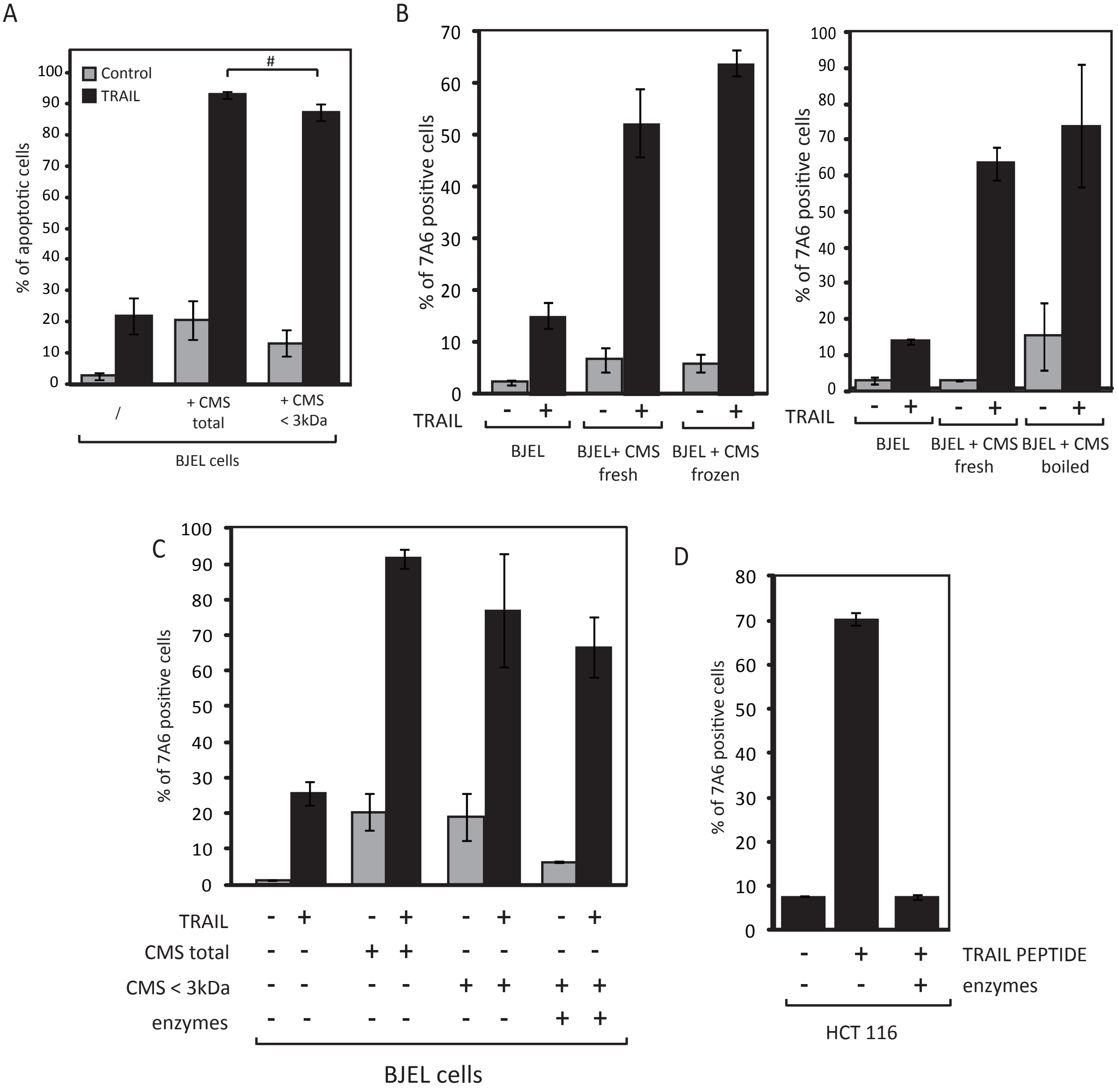

Figure S6. Properties of TRAIL-sensitizing CMS compound(s). **(A)** Both the CMS and the purified < 3 kDa fraction sensitize BJEL cells to comparable levels of TRAIL-induced apoptosis ($n \geq 4$, SEM, # $p > 0.1$). **(B)** CMS factor(s) responsible for sensitization to TRAIL is/are temperature-stable and retain(s) the ability to sensitize BJEL cells to apoptosis after freeze/thawing (left panel) or boiling (right panel). **(C and D)** CMS factor(s) responsible for sensitization retain(s) the ability to sensitize BJEL cells to TRAIL-induced apoptosis after a proteolytic enzyme treatment (C), while the same treatment led to degradation and loss of activity of a synthetic TRAIL peptide (D) (for the TRAIL peptide mimic see Pavet et al. 2010 Cancer Res 70, 1101).
