# Supplementary material for: Senescence-secreted factors activate Myc and sensitize pretransformed cells to TRAIL-induced apoptosis
Source: Aging Cell. 2014 Mar 4;13(3):487–96. doi: 10.1111/acel.12197 (PMC4326894; doi:10.1111/acel.12197)
Supplement: Supplementary file 7 — Fig. S7 Senescent cells enable pretransformed cells to form tumors in nude mice. [file acel0013-0487-sd7.pdf]

A Tumor formation in xenograft experiments:

- HA1ER (transformed) cells	4/4
- HA1E (pre-transformed) cells	0/4
- HA1E + young cells	0/4
- HA1E + senescent cells	3/4

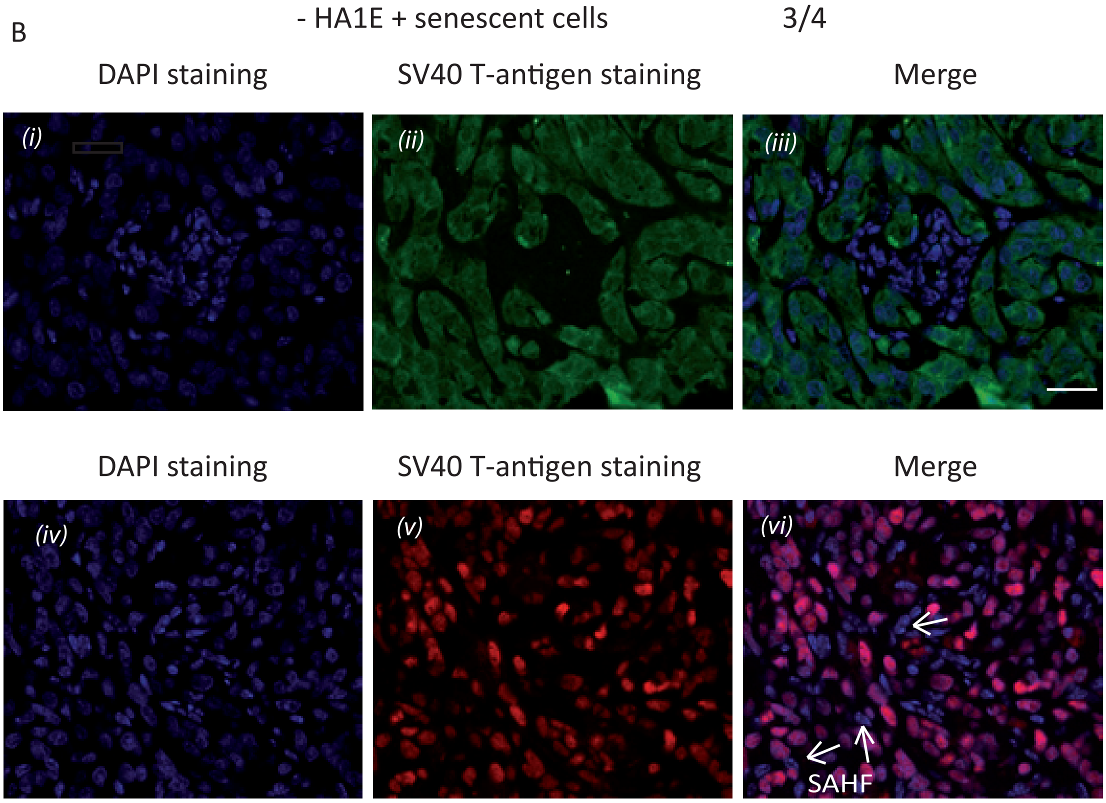

Figure S7. Senescent cells enable pre-transformed cells to form tumors in nude mice.

(A) Representation of tumor formation efficiency in xenograft experiments. Equivalent numbers of either pre-transformed (HA1E) or transformed (HA1ER) cells were injected in nude mice. In co-injection experiments pre-transformed (HA1E) cells were mixed with an equal number of WI-38 young or WI-38 Ras-OIS fibroblasts.

(B) Immunostaining of tumor sections derived from co-injection of HA1E and senescent cells. The majority of the tumor comprises Ki67 positive, replicating cells (*ii*-green cytoplasmatic staining) and SV40 T-antigen positive cells (*v*-red nuclear staining). Senescent cells can be observed as Ki-67, SV40 double-negative cells (*iii*, *vi*), with SAHFs present in the nuclei (*i*, *iii*, *iv*, marked with white arrows in panel *vi*). Bar, 25 µm
