# Supplementary material for: Senescence-secreted factors activate Myc and sensitize pretransformed cells to TRAIL-induced apoptosis
Source: Aging Cell. 2014 Mar 4;13(3):487–96. doi: 10.1111/acel.12197 (PMC4326894; doi:10.1111/acel.12197)
Supplement: Supplementary file 8 — Table S1GO terms associated with paths defined by DREM analysis. [file acel0013-0487-sd8.pdf]

			# Genes						
path	Category ID	Category Name	Category	Assigned	Expected	Enriched	p-value	Corrected p-value	Fold
(i)	GO:0010941	regulation of cell death	1180	52	8,6	43,4	1,40E-27	<0.001	6
(i)	GO:0043067	regulation of programmed cell death	1151	51	8,4	42,6	4,00E-27	<0.001	6
(i)	GO:0012501	programmed cell death	1493	56	10,9	45,1	2,10E-26	<0.001	5,1
(i)	GO:0042981	regulation of apoptotic process	1138	50	8,3	41,7	2,30E-26	<0.001	6
(i)	GO:0008219	cell death	1651	58	12,1	45,9	5,20E-26	<0.001	4,8
(i)	GO:0016265	death	1653	58	12,1	45,9	5,50E-26	<0.001	4,8
(i)	GO:0006915	apoptotic process	1479	55	10,8	44,2	1,10E-25	<0.001	5,1
(i)	GO:0048519	negative regulation of biological process	2919	72	21,4	50,6	6,50E-24	<0.001	3,4
(i)	GO:0048523	negative regulation of cellular process	2667	69	19,5	49,5	7,20E-24	<0.001	3,5
(i)	GO:0048518	positive regulation of biological process	3286	76	24,1	51,9	7,50E-24	<0.001	3,2
(i)	GO:0048522	positive regulation of cellular process	2993	71	21,9	49,1	1,80E-22	<0.001	3,2
(i)	GO:0060548	negative regulation of cell death	590	35	4,3	30,7	2,50E-22	<0.001	8,1
(i)	GO:0006950	response to stress	2762	68	20,2	47,8	3,40E-22	<0.001	3,4
(i)	GO:0043069	negative regulation of programmed cell death	571	34	4,2	29,8	9,80E-22	<0.001	8,1
(i)	GO:0008283	cell proliferation	1475	50	10,8	39,2	2,40E-21	<0.001	4,6
(i)	GO:0042127	regulation of cell proliferation	1131	44	8,3	35,7	6,80E-21	<0.001	5,3
(i)	GO:0043066	negative regulation of apoptotic process	563	33	4,1	28,9	7,00E-21	<0.001	8
(i)	GO:0007243	intracellular protein kinase cascade	856	39	6,3	32,7	8,20E-21	<0.001	6,2
(i)	GO:0010033	response to organic substance	1788	53	13,1	39,9	4,30E-20	<0.001	4
(i)	GO:0033993	response to lipid	515	31	3,8	27,2	6,00E-20	<0.001	8,2
(i)	GO:0007165	signal transduction	3890	77	28,5	48,5	6,10E-20	<0.001	2,7
(i)	GO:0042221	response to chemical stimulus	2611	63	19,1	43,9	9,40E-20	<0.001	3,3
(i)	GO:0071310	cellular response to organic substance	1278	44	9,4	34,6	7,60E-19	<0.001	4,7
(i)	GO:0070887	cellular response to chemical stimulus	1620	49	11,9	37,1	8,80E-19	<0.001	4,1
(i)	GO:0051246	regulation of protein metabolic process	1420	46	10,4	35,6	1,00E-18	<0.001	4,4
(i)	GO:0051716	cellular response to stimulus	4722	83	34,6	48,4	1,10E-18	<0.001	2,4
(i)	GO:0009611	response to wounding	1063	40	7,8	32,2	2,10E-18	<0.001	5,1
(i)	GO:0007154	cell communication	4463	80	32,7	47,3	3,10E-18	<0.001	2,4
(i)	GO:0002237	response to molecule of bacterial origin	208	21	1,5	19,5	3,10E-18	<0.001	13,8
(i)	GO:0044700	single organism signaling	4362	79	32	47	3,40E-18	<0.001	2,5
(i)	GO:0050794	regulation of cellular process	7076	101	51,9	49,1	5,10E-18	<0.001	1,9
(i)	GO:0006952	defense response	1045	39	7,7	31,3	8,40E-18	<0.001	5,1
(i)	GO:0006954	inflammatory response	479	28	3,5	24,5	1,00E-17	<0.001	8
(i)	GO:0009607	response to biotic stimulus	570	30	4,2	25,8	1,10E-17	<0.001	7,2
(i)	GO:0009605	response to external stimulus	1190	41	8,7	32,3	1,70E-17	<0.001	4,7
(i)	GO:0065009	regulation of molecular function	1741	49	12,8	36,2	1,70E-17	<0.001	3,8

(i)	GO:0032496	response to lipopolysaccharide	197	20	1,4	18,6	1,90E-17	<0.001	13,9
(i)	GO:0010627	regulation of intracellular protein kinase cascade	684	32	5	27	2,30E-17	<0.001	6,4
(i)	GO:0044092	negative regulation of molecular function	647	31	4,7	26,3	4,10E-17	<0.001	6,5
(i)	GO:0050789	regulation of biological process	7458	102	54,7	47,3	7,70E-17	<0.001	1,9
(i)	GO:0009617	response to bacterium	321	23	2,4	20,6	1,40E-16	<0.001	9,8
(i)	GO:0009628	response to abiotic stimulus	731	32	5,4	26,6	1,60E-16	<0.001	6
(i)	GO:0051707	response to other organism	546	28	4	24	3,00E-16	<0.001	7
(i)	GO:0048583	regulation of response to stimulus	2196	53	16,1	36,9	3,70E-16	<0.001	3,3
(i)	GO:0006955	immune response	1000	36	7,3	28,7	6,50E-16	<0.001	4,9
(i)	GO:0002682	regulation of immune system process	832	33	6,1	26,9	8,50E-16	<0.001	5,4
(i)	GO:0035556	intracellular signal transduction	1854	48	13,6	34,4	1,10E-15	<0.001	3,5
(i)	GO:0042325	regulation of phosphorylation	841	33	6,2	26,8	1,20E-15	<0.001	5,4
(i)	GO:0001932	regulation of protein phosphorylation	787	32	5,8	26,2	1,30E-15	<0.001	5,5
(i)	GO:0031324	negative regulation of cellular metabolic process	1282	40	9,4	30,6	1,30E-15	<0.001	4,3
(i)	GO:0019222	regulation of metabolic process	4487	76	32,9	43,1	1,60E-15	<0.001	2,3
(i)	GO:0051090	regulation of sequence-specific DNA binding transcription factor activity	324	22	2,4	19,6	2,20E-15	<0.001	9,3
(i)	GO:0005125	cytokine activity	188	18	1,4	16,6	2,40E-15	<0.001	13,1
(i)	GO:0023051	regulation of signaling	1918	48	14,1	33,9	4,30E-15	<0.001	3,4
(i)	GO:0009966	regulation of signal transduction	1690	45	12,4	32,6	4,60E-15	<0.001	3,6
(i)	GO:0010646	regulation of cell communication	1927	48	14,1	33,9	5,20E-15	<0.001	3,4
(i)	GO:0044707	single-multicellular organism process	5039	80	36,9	43,1	5,70E-15	<0.001	2,2
(i)	GO:0060255	regulation of macromolecule metabolic process	3891	69	28,5	40,5	1,10E-14	<0.001	2,4
(i)	GO:0051239	regulation of multicellular organismal process	1742	45	12,8	32,2	1,40E-14	<0.001	3,5
(i)	GO:0080090	regulation of primary metabolic process	4014	70	29,4	40,6	1,40E-14	<0.001	2,4
(i)	GO:0042326	negative regulation of phosphorylation	211	18	1,5	16,5	1,80E-14	<0.001	11,6
(i)	GO:0009892	negative regulation of metabolic process	1386	40	10,2	29,8	1,80E-14	<0.001	3,9
(i)	GO:0050793	regulation of developmental process	1403	40	10,3	29,7	2,70E-14	<0.001	3,9
(i)	GO:0009893	positive regulation of metabolic process	1934	47	14,2	32,8	2,90E-14	<0.001	3,3
(i)	GO:0010563	negative regulation of phosphorus metabolic process	252	19	1,8	17,2	3,10E-14	<0.001	10,3
(i)	GO:0045936	negative regulation of phosphate metabolic process	252	19	1,8	17,2	3,10E-14	<0.001	10,3
(i)	GO:0006468	protein phosphorylation	1078	35	7,9	27,1	4,00E-14	<0.001	4,4
(i)	GO:0016310	phosphorylation	1211	37	8,9	28,1	4,00E-14	<0.001	4,2

(i)	GO:0008285	negative regulation of cell proliferation	510	25	3,7	21,3	4,10E-14	<0.001	6,7
(i)	GO:0048731	system development	3199	61	23,4	37,6	4,70E-14	<0.001	2,6
(i)	GO:0071216	cellular response to biotic stimulus	109	14	0,8	13,2	5,70E-14	<0.001	17,5
(i)	GO:0010605	negative regulation of macromolecule metabolic process	1294	38	9,5	28,5	5,80E-14	<0.001	4
(i)	GO:0032268	regulation of cellular protein metabolic process	1240	37	9,1	27,9	8,40E-14	<0.001	4,1
(i)	GO:0031323	regulation of cellular metabolic process	4071	69	29,8	39,2	1,10E-13	<0.001	2,3
(i)	GO:0008284	positive regulation of cell proliferation	638	27	4,7	22,3	1,20E-13	<0.001	5,8
(i)	GO:0005126	cytokine receptor binding	203	17	1,5	15,5	1,40E-13	<0.001	11,4
(i)	GO:0048513	organ development	2275	50	16,7	33,3	1,50E-13	<0.001	3
(i)	GO:0031399	regulation of protein modification process	999	33	7,3	25,7	1,60E-13	<0.001	4,5
(i)	GO:0007166	cell surface receptor signaling pathway	2197	49	16,1	32,9	1,70E-13	<0.001	3
(i)	GO:0007275	multicellular organismal development	3694	65	27,1	37,9	1,80E-13	<0.001	2,4
(i)	GO:0044763	single-organism cellular process	8856	106	64,9	41,1	2,40E-13	<0.001	1,6
(i)	GO:1901700	response to oxygen-containing compound	958	32	7	25	2,90E-13	<0.001	4,6
(i)	GO:0033554	cellular response to stress	1155	35	8,5	26,5	3,00E-13	<0.001	4,1
(i)	GO:0001817	regulation of cytokine production	370	21	2,7	18,3	3,30E-13	<0.001	7,7
(i)	GO:0001816	cytokine production	416	22	3	19	3,60E-13	<0.001	7,2
(i)	GO:0002684	positive regulation of immune system process	514	24	3,8	20,2	4,00E-13	<0.001	6,4
(i)	GO:0005515	protein binding	6790	91	49,8	41,2	4,20E-13	<0.001	1,8
(i)	GO:0031325	positive regulation of cellular metabolic process	1835	44	13,4	30,6	4,20E-13	<0.001	3,3
(i)	GO:0044767	single-organism developmental process	3263	60	23,9	36,1	4,60E-13	<0.001	2,5
(i)	GO:0002520	immune system development	570	25	4,2	20,8	4,90E-13	<0.001	6
(i)	GO:0048856	anatomical structure development	3671	64	26,9	37,1	5,20E-13	<0.001	2,4
(i)	GO:0009612	response to mechanical stimulus	131	14	1	13	7,60E-13	<0.001	14,6
(i)	GO:0070482	response to oxygen levels	229	17	1,7	15,3	9,80E-13	<0.001	10,1
(i)	GO:0001933	negative regulation of protein phosphorylation	196	16	1,4	14,6	1,10E-12	<0.001	11,1
(i)	GO:0048534	hematopoietic or lymphoid organ development	540	24	4	20	1,20E-12	<0.001	6,1
(i)	GO:0009719	response to endogenous stimulus	1018	32	7,5	24,5	1,50E-12	<0.001	4,3
(i)	GO:0048584	positive regulation of response to stimulus	1091	33	8	25	1,80E-12	<0.001	4,1
(i)	GO:0005615	extracellular space	777	28	5,7	22,3	1,90E-12	<0.001	4,9
(i)	GO:0007167	enzyme linked receptor protein signaling pathway	845	29	6,2	22,8	2,40E-12	<0.001	4,7
(i)	GO:0030097	hemopoiesis	509	23	3,7	19,3	2,60E-12	<0.001	6,2
(i)	GO:0001944	vasculature development	513	23	3,8	19,2	3,00E-12	<0.001	6,1
(i)	GO:0002521	leukocyte differentiation	328	19	2,4	16,6	3,40E-12	<0.001	7,9
(i)	GO:0019220	regulation of phosphate metabolic process	1189	34	8,7	25,3	3,60E-12	<0.001	3,9

(i)	GO:0071222	cellular response to lipopolysaccharide	93	12	0,7	11,3	3,70E-12	<0.001	17,6
(i)	GO:0030099	myeloid cell differentiation	249	17	1,8	15,2	3,80E-12	<0.001	9,3
(i)	GO:0045595	regulation of cell differentiation	991	31	7,3	23,7	4,10E-12	<0.001	4,3
(i)	GO:0008083	growth factor activity	149	14	1,1	12,9	4,50E-12	<0.001	12,8
(i)	GO:0001666	response to hypoxia	215	16	1,6	14,4	4,60E-12	<0.001	10,2
(i)	GO:0010604	positive regulation of macromolecule metabolic process	1804	42	13,2	28,8	4,60E-12	<0.001	3,2
(i)	GO:0051174	regulation of phosphorus metabolic process	1201	34	8,8	25,2	4,80E-12	<0.001	3,9
(i)	GO:0036293	response to decreased oxygen levels	217	16	1,6	14,4	5,30E-12	<0.001	10,1
(i)	GO:0006366	transcription from RNA polymerase II promoter	1365	36	10	26	7,40E-12	<0.001	3,6
(i)	GO:0071219	cellular response to molecule of bacterial origin	99	12	0,7	11,3	7,80E-12	<0.001	16,5
(i)	GO:0001568	blood vessel development	488	22	3,6	18,4	8,50E-12	<0.001	6,2
(i)	GO:0044421	extracellular region part	1027	31	7,5	23,5	1,00E-11	<0.001	4,1
(i)	GO:0080134	regulation of response to stress	715	26	5,2	20,8	1,10E-11	<0.001	5
(i)	GO:0050790	regulation of catalytic activity	1387	36	10,2	25,8	1,20E-11	<0.001	3,5
(i)	GO:0051094	positive regulation of developmental process	668	25	4,9	20,1	1,60E-11	<0.001	5,1
(i)	GO:0051240	positive regulation of multicellular organismal process	505	22	3,7	18,3	1,70E-11	<0.001	5,9
(i)	GO:0051248	negative regulation of protein metabolic process	465	21	3,4	17,6	2,60E-11	<0.001	6,2
(i)	GO:0071396	cellular response to lipid	209	15	1,5	13,5	3,70E-11	<0.001	9,8
(i)	GO:0072358	cardiovascular system development	756	26	5,5	20,5	3,80E-11	<0.001	4,7
(i)	GO:0072359	circulatory system development	756	26	5,5	20,5	3,80E-11	<0.001	4,7
(i)	GO:0051247	positive regulation of protein metabolic process	883	28	6,5	21,5	4,00E-11	<0.001	4,3
(i)	GO:2000026	regulation of multicellular organismal development	1089	31	8	23	4,50E-11	<0.001	3,9
(i)	GO:0051171	regulation of nitrogen compound metabolic process	3223	56	23,6	32,4	4,70E-11	<0.001	2,4
(i)	GO:0010942	positive regulation of cell death	435	20	3,2	16,8	5,80E-11	<0.001	6,3
(i)	GO:0045859	regulation of protein kinase activity	596	23	4,4	18,6	6,20E-11	<0.001	5,3
(i)	GO:0010741	negative regulation of intracellular protein kinase cascade	148	13	1,1	11,9	6,40E-11	<0.001	12
(i)	GO:0019219	regulation of nucleobase-containing compound metabolic process	3149	55	23,1	31,9	6,50E-11	<0.001	2,4
(i)	GO:0048660	regulation of smooth muscle cell proliferation	69	10	0,5	9,5	7,50E-11	<0.001	19,8
(i)	GO:0014070	response to organic cyclic compound	494	21	3,6	17,4	7,90E-11	<0.001	5,8
(i)	GO:0006357	regulation of transcription from RNA polymerase II promoter	1185	32	8,7	23,3	7,90E-11	<0.001	3,7

(i)	GO:0006469	negative regulation of protein kinase activity	151	13	1,1	11,9	8,30E-11	<0.001	11,7
(i)	GO:0043086	negative regulation of catalytic activity	502	21	3,7	17,3	1,10E-10	<0.001	5,7
(i)	GO:0048659	smooth muscle cell proliferation	72	10	0,5	9,5	1,20E-10	<0.001	19
(i)	GO:0032269	negative regulation of cellular protein metabolic process	408	19	3	16	1,50E-10	<0.001	6,4
(i)	GO:0043549	regulation of kinase activity	623	23	4,6	18,4	1,50E-10	<0.001	5
(i)	GO:0033673	negative regulation of kinase activity	161	13	1,2	11,8	1,90E-10	<0.001	11
(i)	GO:0031347	regulation of defense response	414	19	3	16	1,90E-10	<0.001	6,3
(i)	GO:0010556	regulation of macromolecule biosynthetic process	2948	52	21,6	30,4	2,10E-10	<0.001	2,4
(i)	GO:0051173	positive regulation of nitrogen compound metabolic process	1160	31	8,5	22,5	2,20E-10	<0.001	3,6
(i)	GO:0002573	myeloid leukocyte differentiation	132	12	1	11	2,40E-10	<0.001	12,4
(i)	GO:0043068	positive regulation of programmed cell death	420	19	3,1	15,9	2,40E-10	<0.001	6,2
(i)	GO:0009653	anatomical structure morphogenesis	1962	41	14,4	26,6	2,70E-10	<0.001	2,9
(i)	GO:2000112	regulation of cellular macromolecule biosynthetic process	2876	51	21,1	29,9	2,80E-10	<0.001	2,4
(i)	GO:0051338	regulation of transferase activity	644	23	4,7	18,3	2,90E-10	<0.001	4,9
(i)	GO:0048514	blood vessel morphogenesis	427	19	3,1	15,9	3,20E-10	<0.001	6,1
(i)	GO:0042493	response to drug	332	17	2,4	14,6	3,50E-10	<0.001	7
(i)	GO:0009889	regulation of biosynthetic process	3091	53	22,7	30,3	3,60E-10	<0.001	2,3
(i)	GO:0051348	negative regulation of transferase activity	172	13	1,3	11,7	4,20E-10	<0.001	10,3
(i)	GO:0048661	positive regulation of smooth muscle cell proliferation	41	8	0,3	7,7	5,30E-10	<0.001	26,6
(i)	GO:0065008	regulation of biological quality	2462	46	18	28	6,00E-10	<0.001	2,5
(i)	GO:0043433	negative regulation of sequence-specific DNA binding transcription factor activity	112	11	0,8	10,2	6,10E-10	<0.001	13,4
(i)	GO:0002224	toll-like receptor signaling pathway	113	11	0,8	10,2	6,70E-10	<0.001	13,3
(i)	GO:0045429	positive regulation of nitric oxide biosynthetic process	27	7	0,2	6,8	7,60E-10	<0.001	35,4
(i)	GO:0031326	regulation of cellular biosynthetic process	3065	52	22,5	29,5	8,60E-10	<0.001	2,3
(i)	GO:0001525	angiogenesis	354	17	2,6	14,4	9,30E-10	<0.001	6,6
(i)	GO:0048585	negative regulation of response to stimulus	748	24	5,5	18,5	9,60E-10	<0.001	4,4
(i)	GO:0023056	positive regulation of signaling	814	25	6	19	1,00E-09	<0.001	4,2
(i)	GO:0071495	cellular response to endogenous stimulus	688	23	5	18	1,00E-09	<0.001	4,6
(i)	GO:0010647	positive regulation of cell communication	816	25	6	19	1,10E-09	<0.001	4,2
(i)	GO:0009059	macromolecule biosynthetic process	3909	60	28,6	31,4	1,10E-09	<0.001	2,1

(i)	GO:0044271	cellular nitrogen compound biosynthetic process	3387	55	24,8	30,2	1,10E-09	<0.001	2,2
(i)	GO:0009891	positive regulation of biosynthetic process	1239	31	9,1	21,9	1,10E-09	<0.001	3,4
(i)	GO:0031349	positive regulation of defense response	225	14	1,6	12,4	1,10E-09	<0.001	8,5
(i)	GO:0000165	MAPK cascade	516	20	3,8	16,2	1,20E-09	<0.001	5,3
(i)	GO:0031400	negative regulation of protein modification process	315	16	2,3	13,7	1,30E-09	<0.001	6,9
(i)	GO:0043065	positive regulation of apoptotic process	414	18	3	15	1,40E-09	<0.001	5,9
(i)	GO:0006351	transcription, DNA-dependent	2914	50	21,4	28,6	1,50E-09	<0.001	2,3
(i)	GO:0030154	cell differentiation	2538	46	18,6	27,4	1,60E-09	<0.001	2,5
(i)	GO:0014074	response to purine-containing compound	94	10	0,7	9,3	1,70E-09	<0.001	14,5
(i)	GO:0006355	regulation of transcription, DNA-dependent	2639	47	19,3	27,7	1,80E-09	<0.001	2,4
(i)	GO:2001141	regulation of RNA biosynthetic process	2657	47	19,5	27,5	2,20E-09	<0.001	2,4
(i)	GO:0010628	positive regulation of gene expression	1054	28	7,7	20,3	2,20E-09	<0.001	3,6
(i)	GO:0045321	leukocyte activation	539	20	4	16	2,50E-09	<0.001	5,1
(i)	GO:0009888	tissue development	1211	30	8,9	21,1	2,70E-09	<0.001	3,4
(i)	GO:0002221	pattern recognition receptor signaling pathway	129	11	0,9	10,1	2,80E-09	<0.001	11,6
(i)	GO:1901701	cellular response to oxygen-containing compound	603	21	4,4	16,6	2,90E-09	<0.001	4,8
(i)	GO:0031328	positive regulation of cellular biosynthetic process	1220	30	8,9	21,1	3,20E-09	<0.001	3,4
(i)	GO:0048545	response to steroid hormone stimulus	245	14	1,8	12,2	3,40E-09	<0.001	7,8
(i)	GO:0002758	innate immune response-activating signal transduction	132	11	1	10	3,50E-09	<0.001	11,4
(i)	GO:0048869	cellular developmental process	2697	47	19,8	27,2	3,60E-09	<0.001	2,4
(i)	GO:0032774	RNA biosynthetic process	2990	50	21,9	28,1	3,60E-09	<0.001	2,3
(i)	GO:0010468	regulation of gene expression	3095	51	22,7	28,3	3,90E-09	<0.001	2,2
(i)	GO:0097305	response to alcohol	207	13	1,5	11,5	4,10E-09	<0.001	8,6
(i)	GO:0051252	regulation of RNA metabolic process	2712	47	19,9	27,1	4,30E-09	<0.001	2,4
(i)	GO:0010038	response to metal ion	209	13	1,5	11,5	4,60E-09	<0.001	8,5
(i)	GO:0007249	I-kappaB kinase/NF-kappaB cascade	212	13	1,6	11,4	5,50E-09	<0.001	8,4
(i)	GO:0031663	lipopolysaccharide-mediated signaling pathway	35	7	0,3	6,7	5,50E-09	<0.001	27,3
(i)	GO:0009314	response to radiation	300	15	2,2	12,8	5,60E-09	<0.001	6,8
(i)	GO:0043170	macromolecule metabolic process	6694	82	49,1	32,9	5,90E-09	<0.001	1,7
(i)	GO:0002218	activation of innate immune response	140	11	1	10	6,60E-09	<0.001	10,7
(i)	GO:0050776	regulation of immune response	513	19	3,8	15,2	6,60E-09	<0.001	5,1
(i)	GO:0033002	muscle cell proliferation	108	10	0,8	9,2	6,70E-09	<0.001	12,6

(i)	GO:0034654	nucleobase-containing compound biosynthetic process	3253	52	23,8	28,2	7,30E-09	<0.001	2,2
(i)	GO:0051091	positive regulation of sequence-specific DNA binding transcription factor activity	178	12	1,3	10,7	7,50E-09	<0.001	9,2
(i)	GO:0045428	regulation of nitric oxide biosynthetic process	37	7	0,3	6,7	8,30E-09	<0.001	25,8
(i)	GO:0023057	negative regulation of signaling	642	21	4,7	16,3	8,80E-09	<0.001	4,5
(i)	GO:0010648	negative regulation of cell communication	646	21	4,7	16,3	9,80E-09	<0.001	4,4
(i)	GO:0051051	negative regulation of transport	269	14	2	12	1,10E-08	<0.001	7,1
(i)	GO:0071900	regulation of protein serine/threonine kinase activity	367	16	2,7	13,3	1,20E-08	<0.001	5,9
(i)	GO:0046683	response to organophosphorus	85	9	0,6	8,4	1,20E-08	<0.001	14,4
(i)	GO:0034097	response to cytokine stimulus	476	18	3,5	14,5	1,20E-08	<0.001	5,2
(i)	GO:0001893	maternal placenta development	23	6	0,2	5,8	1,30E-08	<0.001	35,6
(i)	GO:0019438	aromatic compound biosynthetic process	3306	52	24,2	27,8	1,30E-08	<0.001	2,1
(i)	GO:0071496	cellular response to external stimulus	187	12	1,4	10,6	1,30E-08	<0.001	8,8
(i)	GO:0019538	protein metabolic process	3624	55	26,6	28,4	1,30E-08	<0.001	2,1
(i)	GO:0018130	heterocycle biosynthetic process	3313	52	24,3	27,7	1,40E-08	<0.001	2,1
(i)	GO:0010557	positive regulation of macromolecule biosynthetic process	1146	28	8,4	19,6	1,40E-08	<0.001	3,3
(i)	GO:0034142	toll-like receptor 4 signaling pathway	87	9	0,6	8,4	1,50E-08	<0.001	14,1
(i)	GO:0010740	positive regulation of intracellular protein kinase cascade	482	18	3,5	14,5	1,50E-08	<0.001	5,1
(i)	GO:0005102	receptor binding	1077	27	7,9	19,1	1,50E-08	<0.001	3,4
(i)	GO:0032879	regulation of localization	1311	30	9,6	20,4	1,70E-08	<0.001	3,1
(i)	GO:0045597	positive regulation of cell differentiation	488	18	3,6	14,4	1,80E-08	<0.001	5
(i)	GO:0001775	cell activation	738	22	5,4	16,6	2,00E-08	<0.001	4,1
(i)	GO:0001934	positive regulation of protein phosphorylation	550	19	4	15	2,00E-08	<0.001	4,7
(i)	GO:0009968	negative regulation of signal transduction	613	20	4,5	15,5	2,10E-08	<0.001	4,5
(i)	GO:0001819	positive regulation of cytokine production	196	12	1,4	10,6	2,20E-08	<0.001	8,4
(i)	GO:0034504	protein localization to nucleus	240	13	1,8	11,2	2,40E-08	<0.001	7,4
(i)	GO:0071901	negative regulation of protein serine/threonine kinase activity	92	9	0,7	8,3	2,40E-08	<0.001	13,3
(i)	GO:0048646	anatomical structure formation involved in morphogenesis	815	23	6	17	2,50E-08	<0.001	3,9
(i)	GO:0034645	cellular macromolecule biosynthetic process	3798	56	27,8	28,2	2,60E-08	<0.001	2
(i)	GO:0045944	positive regulation of transcription from RNA polymerase II promoter	684	21	5	16	2,60E-08	<0.001	4,2
(i)	GO:0042035	regulation of cytokine biosynthetic process	93	9	0,7	8,3	2,70E-08	<0.001	13,2

(i)	GO:0042327	positive regulation of phosphorylation	561	19	4,1	14,9	2,80E-08	<0.001	4,6
(i)	GO:0009725	response to hormone stimulus	628	20	4,6	15,4	3,20E-08	<0.001	4,3
(i)	GO:0043408	regulation of MAPK cascade	451	17	3,3	13,7	3,40E-08	<0.001	5,1
(i)	GO:0010243	response to organic nitrogen	570	19	4,2	14,8	3,60E-08	<0.001	4,5
(i)	GO:0045089	positive regulation of innate immune response	165	11	1,2	9,8	3,70E-08	<0.001	9,1
(i)	GO:1901362	organic cyclic compound biosynthetic process	3410	52	25	27	3,70E-08	<0.001	2,1
(i)	GO:0002756	MyD88-independent toll-like receptor signaling pathway	69	8	0,5	7,5	3,90E-08	<0.001	15,8
(i)	GO:0010035	response to inorganic substance	297	14	2,2	11,8	3,90E-08	<0.001	6,4
(i)	GO:0044744	protein targeting to nucleus	207	12	1,5	10,5	4,10E-08	<0.001	7,9
(i)	GO:0006606	protein import into nucleus	207	12	1,5	10,5	4,10E-08	<0.001	7,9
(i)	GO:1900180	regulation of protein localization to nucleus	131	10	1	9	4,30E-08	<0.001	10,4
(i)	GO:0034134	toll-like receptor 2 signaling pathway	70	8	0,5	7,5	4,30E-08	<0.001	15,6
(i)	GO:0044260	cellular macromolecule metabolic process	6062	75	44,4	30,6	4,40E-08	<0.001	1,7
(i)	GO:0009967	positive regulation of signal transduction	772	22	5,7	16,3	4,40E-08	<0.001	3,9
(i)	GO:1901342	regulation of vasculature development	169	11	1,2	9,8	4,70E-08	<0.001	8,9
(i)	GO:0051170	nuclear import	210	12	1,5	10,5	4,80E-08	<0.001	7,8
(i)	GO:0006809	nitric oxide biosynthetic process	47	7	0,3	6,7	4,80E-08	<0.001	20,3
(i)	GO:0045935	positive regulation of nucleobase-containing compound metabolic process	1141	27	8,4	18,6	5,10E-08	<0.001	3,2
(i)	GO:0046697	decidualization	15	5	0,1	4,9	5,60E-08	<0.001	45,5
(i)	GO:0006464	cellular protein modification process	2356	41	17,3	23,7	5,80E-08	<0.001	2,4
(i)	GO:0036211	protein modification process	2356	41	17,3	23,7	5,80E-08	<0.001	2,4
(i)	GO:0043412	macromolecule modification	2454	42	18	24	6,00E-08	<0.001	2,3
(i)	GO:0022603	regulation of anatomical structure morphogenesis	591	19	4,3	14,7	6,30E-08	<0.001	4,4
(i)	GO:0044093	positive regulation of molecular function	1077	26	7,9	18,1	6,40E-08	<0.001	3,3
(i)	GO:0051092	positive regulation of NF-kappaB transcription factor activity	103	9	0,8	8,2	6,50E-08	<0.001	11,9
(i)	GO:0034138	toll-like receptor 3 signaling pathway	74	8	0,5	7,5	6,80E-08	<0.001	14,8
(i)	GO:0045088	regulation of innate immune response	217	12	1,6	10,4	6,80E-08	<0.001	7,5
(i)	GO:0051384	response to glucocorticoid stimulus	104	9	0,8	8,2	7,10E-08	<0.001	11,8
(i)	GO:0042089	cytokine biosynthetic process	104	9	0,8	8,2	7,10E-08	<0.001	11,8
(i)	GO:0071363	cellular response to growth factor stimulus	421	16	3,1	12,9	8,00E-08	<0.001	5,2
(i)	GO:0043409	negative regulation of MAPK cascade	106	9	0,8	8,2	8,40E-08	<0.001	11,6
(i)	GO:0051254	positive regulation of RNA metabolic process	1018	25	7,5	17,5	8,60E-08	<0.001	3,4

(i)	GO:0032270	positive regulation of cellular protein metabolic process	802	22	5,9	16,1	8,60E-08	<0.001	3,7
(i)	GO:1901698	response to nitrogen compound	603	19	4,4	14,6	8,70E-08	<0.001	4,3
(i)	GO:0042107	cytokine metabolic process	107	9	0,8	8,2	9,10E-08	<0.001	11,5
(i)	GO:0044706	multi-multicellular organism process	182	11	1,3	9,7	1,00E-07	<0.001	8,2
(i)	GO:0045596	negative regulation of cell differentiation	431	16	3,2	12,8	1,10E-07	<0.001	5,1
(i)	GO:0032101	regulation of response to external stimulus	376	15	2,8	12,2	1,10E-07	<0.001	5,4
(i)	GO:0002755	MyD88-dependent toll-like receptor signaling pathway	79	8	0,6	7,4	1,10E-07	<0.001	13,8
(i)	GO:0031960	response to corticosteroid stimulus	110	9	0,8	8,2	1,20E-07	<0.001	11,2
(i)	GO:0070848	response to growth factor stimulus	433	16	3,2	12,8	1,20E-07	<0.001	5
(i)	GO:0050673	epithelial cell proliferation	229	12	1,7	10,3	1,20E-07	<0.001	7,1
(i)	GO:0045087	innate immune response	493	17	3,6	13,4	1,20E-07	<0.001	4,7
(i)	GO:0016477	cell migration	821	22	6	16	1,30E-07	0,002	3,7
(i)	GO:0045893	positive regulation of transcription, DNA-dependent	973	24	7,1	16,9	1,50E-07	0,002	3,4
(i)	GO:0071456	cellular response to hypoxia	82	8	0,6	7,4	1,50E-07	0,002	13,3
(i)	GO:0036294	cellular response to decreased oxygen levels	82	8	0,6	7,4	1,50E-07	0,002	13,3
(i)	GO:0043122	regulation of I-kappaB kinase/NF-kappaB cascade	191	11	1,4	9,6	1,60E-07	0,002	7,9
(i)	GO:0050864	regulation of B cell activation	83	8	0,6	7,4	1,70E-07	0,002	13,2
(i)	GO:0009991	response to extracellular stimulus	285	13	2,1	10,9	1,80E-07	0,002	6,2
(i)	GO:0048568	embryonic organ development	337	14	2,5	11,5	1,90E-07	0,002	5,7
(i)	GO:0010562	positive regulation of phosphorus metabolic process	635	19	4,7	14,3	1,90E-07	0,002	4,1
(i)	GO:0045937	positive regulation of phosphate metabolic process	635	19	4,7	14,3	1,90E-07	0,002	4,1
(i)	GO:0017038	protein import	239	12	1,8	10,2	2,00E-07	0,002	6,9
(i)	GO:0010467	gene expression	4026	56	29,5	26,5	2,10E-07	0,002	1,9
(i)	GO:0045765	regulation of angiogenesis	155	10	1,1	8,9	2,10E-07	0,002	8,8
(i)	GO:0046649	lymphocyte activation	455	16	3,3	12,7	2,30E-07	0,002	4,8
(i)	GO:1901214	regulation of neuron death	157	10	1,2	8,8	2,40E-07	0,002	8,7
(i)	GO:0002764	immune response-regulating signaling pathway	245	12	1,8	10,2	2,50E-07	0,002	6,7
(i)	GO:0046209	nitric oxide metabolic process	60	7	0,4	6,6	2,70E-07	0,002	15,9
(i)	GO:0071453	cellular response to oxygen levels	89	8	0,7	7,3	2,90E-07	0,002	12,3
(i)	GO:0006796	phosphate-containing compound metabolic process	2306	39	16,9	22,1	3,00E-07	0,002	2,3
(i)	GO:0000302	response to reactive oxygen species	123	9	0,9	8,1	3,00E-07	0,002	10

(i)	GO:0043405	regulation of MAP kinase activity	249	12	1,8	10,2	3,00E-07	0,002	6,6
(i)	GO:0071347	cellular response to interleukin-1	38	6	0,3	5,7	3,20E-07	0,002	21,5
(i)	GO:0034641	cellular nitrogen compound metabolic process	5109	65	37,4	27,6	3,30E-07	0,002	1,7
(i)	GO:0051241	negative regulation of multicellular organismal process	353	14	2,6	11,4	3,30E-07	0,002	5,4
(i)	GO:0051591	response to cAMP	62	7	0,5	6,5	3,40E-07	0,002	15,4
(i)	GO:0034101	erythrocyte homeostasis	91	8	0,7	7,3	3,40E-07	0,002	12
(i)	GO:0031327	negative regulation of cellular biosynthetic process	943	23	6,9	16,1	3,40E-07	0,002	3,3
(i)	GO:0051336	regulation of hydrolase activity	731	20	5,4	14,6	3,70E-07	0,002	3,7
(i)	GO:0007179	transforming growth factor beta receptor signaling pathway	165	10	1,2	8,8	3,80E-07	0,002	8,3
(i)	GO:0016070	RNA metabolic process	3553	51	26	25	4,00E-07	0,002	2
(i)	GO:0042306	regulation of protein import into nucleus	127	9	0,9	8,1	4,00E-07	0,002	9,7
(i)	GO:0051093	negative regulation of developmental process	536	17	3,9	13,1	4,00E-07	0,002	4,3
(i)	GO:0042113	B cell activation	166	10	1,2	8,8	4,00E-07	0,002	8,2
(i)	GO:0009890	negative regulation of biosynthetic process	954	23	7	16	4,20E-07	0,002	3,3
(i)	GO:0033135	regulation of peptidyl-serine phosphorylation	64	7	0,5	6,5	4,30E-07	0,002	14,9
(i)	GO:0043407	negative regulation of MAP kinase activity	64	7	0,5	6,5	4,30E-07	0,002	14,9
(i)	GO:0010574	regulation of vascular endothelial growth factor production	22	5	0,2	4,8	4,70E-07	0,002	31
(i)	GO:0010573	vascular endothelial growth factor production	22	5	0,2	4,8	4,70E-07	0,002	31
(i)	GO:0051049	regulation of transport	961	23	7	16	4,80E-07	0,002	3,3
(i)	GO:0007178	transmembrane receptor protein serine/threonine kinase signaling pathway	260	12	1,9	10,1	4,80E-07	0,002	6,3
(i)	GO:0006793	phosphorus metabolic process	2353	39	17,2	21,8	5,00E-07	0,002	2,3
(i)	GO:1901576	organic substance biosynthetic process	4696	61	34,4	26,6	5,00E-07	0,002	1,8
(i)	GO:0048870	cell motility	891	22	6,5	15,5	5,20E-07	0,002	3,4
(i)	GO:0051674	localization of cell	891	22	6,5	15,5	5,20E-07	0,002	3,4
(i)	GO:0003700	sequence-specific DNA binding transcription factor activity	892	22	6,5	15,5	5,30E-07	0,002	3,4
(i)	GO:0001890	placenta development	132	9	1	8	5,50E-07	0,002	9,3
(i)	GO:0048872	homeostasis of number of cells	172	10	1,3	8,7	5,60E-07	0,002	7,9
(i)	GO:0070851	growth factor receptor binding	97	8	0,7	7,3	5,60E-07	0,002	11,3
(i)	GO:0008009	chemokine activity	42	6	0,3	5,7	5,90E-07	0,002	19,5
(i)	GO:0035666	TRIF-dependent toll-like receptor signaling pathway	67	7	0,5	6,5	5,90E-07	0,002	14,3
(i)	GO:0034130	toll-like receptor 1 signaling pathway	67	7	0,5	6,5	5,90E-07	0,002	14,3
(i)	GO:0045637	regulation of myeloid cell differentiation	133	9	1	8	5,90E-07	0,002	9,2

(i)	GO:0010558	negative regulation of macromolecule biosynthetic process	902	22	6,6	15,4	6,40E-07	0,002	3,3
(i)	GO:0042592	homeostatic process	1215	26	8,9	17,1	6,60E-07	0,002	2,9
(i)	GO:0019221	cytokine-mediated signaling pathway	320	13	2,3	10,7	6,70E-07	0,002	5,5
(i)	GO:0060135	maternal process involved in female pregnancy	43	6	0,3	5,7	6,80E-07	0,002	19
(i)	GO:0090304	nucleic acid metabolic process	4054	55	29,7	25,3	6,90E-07	0,002	1,9
(i)	GO:0070997	neuron death	176	10	1,3	8,7	6,90E-07	0,002	7,8
(i)	GO:0002694	regulation of leukocyte activation	324	13	2,4	10,6	7,70E-07	0,002	5,5
(i)	GO:0046888	negative regulation of hormone secretion	44	6	0,3	5,7	7,80E-07	0,002	18,6
(i)	GO:0035872	nucleotide-binding domain, leucine rich repeat containing receptor signaling pathway	44	6	0,3	5,7	7,80E-07	0,002	18,6
(i)	GO:0051726	regulation of cell cycle	628	18	4,6	13,4	7,80E-07	0,002	3,9
(i)	GO:0009058	biosynthetic process	4754	61	34,8	26,2	7,90E-07	0,002	1,8
(i)	GO:0006139	nucleobase-containing compound metabolic process	4759	61	34,9	26,1	8,20E-07	0,002	1,7
(i)	GO:0097285	cell-type specific apoptotic process	274	12	2	10	8,40E-07	0,002	6
(i)	GO:0051346	negative regulation of hydrolase activity	226	11	1,7	9,3	8,80E-07	0,002	6,6
(i)	GO:0050869	negative regulation of B cell activation	25	5	0,2	4,8	9,30E-07	0,002	27,3
(i)	GO:0002695	negative regulation of leukocyte activation	104	8	0,8	7,2	9,60E-07	0,002	10,5
(i)	GO:0007568	aging	184	10	1,3	8,7	1,00E-06	0,002	7,4
(i)	GO:1901654	response to ketone	73	7	0,5	6,5	1,10E-06	0,002	13,1
(i)	GO:0002757	immune response-activating signal transduction	233	11	1,7	9,3	1,20E-06	0,002	6,4
(i)	GO:0031401	positive regulation of protein modification process	716	19	5,2	13,8	1,20E-06	0,002	3,6
(i)	GO:0070661	leukocyte proliferation	187	10	1,4	8,6	1,20E-06	0,002	7,3
(i)	GO:0044237	cellular metabolic process	7838	85	57,4	27,6	1,20E-06	0,002	1,5
(i)	GO:0071345	cellular response to cytokine stimulus	396	14	2,9	11,1	1,30E-06	0,002	4,8
(i)	GO:0051249	regulation of lymphocyte activation	286	12	2,1	9,9	1,30E-06	0,002	5,7
(i)	GO:0060341	regulation of cellular localization	652	18	4,8	13,2	1,30E-06	0,002	3,8
(i)	GO:0030183	B cell differentiation	76	7	0,6	6,4	1,40E-06	0,002	12,6
(i)	GO:0071560	cellular response to transforming growth factor beta stimulus	192	10	1,4	8,6	1,50E-06	0,002	7,1
(i)	GO:0070663	regulation of leukocyte proliferation	149	9	1,1	7,9	1,50E-06	0,002	8,2
(i)	GO:0052548	regulation of endopeptidase activity	240	11	1,8	9,2	1,60E-06	0,002	6,3
(i)	GO:0006807	nitrogen compound metabolic process	5442	66	39,9	26,1	1,60E-06	0,002	1,7
(i)	GO:0071559	response to transforming growth factor beta stimulus	193	10	1,4	8,6	1,60E-06	0,002	7,1

(i)	GO:0033138	positive regulation of peptidyl-serine phosphorylation	50	6	0,4	5,6	1,70E-06	0,002	16,4
(i)	GO:0044249	cellular biosynthetic process	4623	59	33,9	25,1	1,70E-06	0,002	1,7
(i)	GO:0007169	transmembrane receptor protein tyrosine kinase signaling pathway	596	17	4,4	12,6	1,70E-06	0,002	3,9
(i)	GO:0050865	regulation of cell activation	349	13	2,6	10,4	1,80E-06	0,002	5,1
(i)	GO:0050866	negative regulation of cell activation	113	8	0,8	7,2	1,80E-06	0,002	9,7
(i)	GO:0051050	positive regulation of transport	470	15	3,4	11,6	1,90E-06	0,002	4,4
(i)	GO:0042379	chemokine receptor binding	51	6	0,4	5,6	1,90E-06	0,002	16,1
(i)	GO:0008063	Toll signaling pathway	80	7	0,6	6,4	2,00E-06	0,002	11,9
(i)	GO:0006979	response to oxidative stress	246	11	1,8	9,2	2,00E-06	0,002	6,1
(i)	GO:0046822	regulation of nucleocytoplasmic transport	154	9	1,1	7,9	2,00E-06	0,002	8
(i)	GO:0052547	regulation of peptidase activity	248	11	1,8	9,2	2,20E-06	0,002	6,1
(i)	GO:0050679	positive regulation of epithelial cell proliferation	116	8	0,9	7,1	2,20E-06	0,002	9,4
(i)	GO:0046483	heterocycle metabolic process	4894	61	35,9	25,1	2,20E-06	0,002	1,7
(i)	GO:0042542	response to hydrogen peroxide	82	7	0,6	6,4	2,30E-06	0,002	11,6
(i)	GO:0006725	cellular aromatic compound metabolic process	4900	61	35,9	25,1	2,30E-06	0,002	1,7
(i)	GO:0044267	cellular protein metabolic process	3015	44	22,1	21,9	2,40E-06	0,002	2
(i)	GO:0050731	positive regulation of peptidyl-tyrosine phosphorylation	118	8	0,9	7,1	2,50E-06	0,002	9,3
(i)	GO:0000122	negative regulation of transcription from RNA polymerase II promoter	483	15	3,5	11,5	2,60E-06	0,002	4,2
(i)	GO:0001892	embryonic placenta development	84	7	0,6	6,4	2,70E-06	0,002	11,4
(i)	GO:0032386	regulation of intracellular transport	255	11	1,9	9,1	2,80E-06	0,002	5,9
(i)	GO:0071260	cellular response to mechanical stimulus	55	6	0,4	5,6	3,00E-06	0,002	14,9
(i)	GO:0051048	negative regulation of secretion	121	8	0,9	7,1	3,00E-06	0,002	9
(i)	GO:0010951	negative regulation of endopeptidase activity	121	8	0,9	7,1	3,00E-06	0,002	9
(i)	GO:0050900	leukocyte migration	257	11	1,9	9,1	3,10E-06	0,002	5,8
(i)	GO:0006928	cellular component movement	1153	24	8,5	15,5	3,10E-06	0,002	2,8
(i)	GO:0030218	erythrocyte differentiation	86	7	0,6	6,4	3,20E-06	0,004	11,1
(i)	GO:0002685	regulation of leukocyte migration	86	7	0,6	6,4	3,20E-06	0,004	11,1
(i)	GO:0002252	immune effector process	430	14	3,2	10,8	3,40E-06	0,004	4,4
(i)	GO:0045892	negative regulation of transcription, DNA-dependent	770	19	5,6	13,4	3,40E-06	0,004	3,4
(i)	GO:0031667	response to nutrient levels	261	11	1,9	9,1	3,60E-06	0,004	5,8

(i)	GO:0010466	negative regulation of peptidase activity	124	8	0,9	7,1	3,60E-06	0,004	8,8
(i)	GO:0043154	negative regulation of cysteine-type endopeptidase activity involved in apoptotic process	57	6	0,4	5,6	3,70E-06	0,004	14,4
(i)	GO:0033157	regulation of intracellular protein transport	167	9	1,2	7,8	3,90E-06	0,004	7,4
(i)	GO:0010629	negative regulation of gene expression	855	20	6,3	13,7	4,20E-06	0,004	3,2
(i)	GO:0043392	negative regulation of DNA binding	34	5	0,2	4,8	4,60E-06	0,004	20,1
(i)	GO:0071704	organic substance metabolic process	8192	86	60	26	4,60E-06	0,004	1,4
(i)	GO:0032844	regulation of homeostatic process	270	11	2	9	4,90E-06	0,004	5,6
(i)	GO:2000116	regulation of cysteine-type endopeptidase activity	172	9	1,3	7,7	5,00E-06	0,004	7,1
(i)	GO:2000117	negative regulation of cysteine-type endopeptidase activity	60	6	0,4	5,6	5,00E-06	0,004	13,6
(i)	GO:0070555	response to interleukin-1	60	6	0,4	5,6	5,00E-06	0,004	13,6
(i)	GO:0051250	negative regulation of lymphocyte activation	92	7	0,7	6,3	5,10E-06	0,004	10,4
(i)	GO:2000113	negative regulation of cellular macromolecule biosynthetic process	868	20	6,4	13,6	5,20E-06	0,004	3,1
(i)	GO:0002683	negative regulation of immune system process	173	9	1,3	7,7	5,20E-06	0,004	7,1
(i)	GO:0030334	regulation of cell migration	386	13	2,8	10,2	5,30E-06	0,004	4,6
(i)	GO:0045639	positive regulation of myeloid cell differentiation	61	6	0,4	5,6	5,50E-06	0,004	13,4
(i)	GO:0051253	negative regulation of RNA metabolic process	798	19	5,8	13,2	5,80E-06	0,004	3,2
(i)	GO:0045444	fat cell differentiation	132	8	1	7	5,80E-06	0,004	8,3
(i)	GO:0051128	regulation of cellular component organization	1197	24	8,8	15,2	5,90E-06	0,004	2,7
(i)	GO:0006913	nucleocytoplasmic transport	332	12	2,4	9,6	6,10E-06	0,004	4,9
(i)	GO:0044238	primary metabolic process	7967	84	58,4	25,6	6,10E-06	0,004	1,4
(i)	GO:0002687	positive regulation of leukocyte migration	63	6	0,5	5,5	6,70E-06	0,004	13
(i)	GO:0002253	activation of immune response	279	11	2	9	6,70E-06	0,004	5,4
(i)	GO:0051169	nuclear transport	335	12	2,5	9,5	6,70E-06	0,004	4,9
(i)	GO:0030098	lymphocyte differentiation	227	10	1,7	8,3	6,80E-06	0,004	6
(i)	GO:0051272	positive regulation of cellular component movement	228	10	1,7	8,3	7,10E-06	0,004	6
(i)	GO:0051270	regulation of cellular component movement	459	14	3,4	10,6	7,10E-06	0,004	4,2
(i)	GO:0042990	regulation of transcription factor import into nucleus	64	6	0,5	5,5	7,30E-06	0,004	12,8
(i)	GO:0009790	embryo development	889	20	6,5	13,5	7,40E-06	0,004	3,1
(i)	GO:0042991	transcription factor import into nucleus	65	6	0,5	5,5	8,00E-06	0,004	12,6
(i)	GO:0051223	regulation of protein transport	286	11	2,1	8,9	8,50E-06	0,004	5,2

(i)	GO:0050778	positive regulation of immune response	344	12	2,5	9,5	8,80E-06	0,004	4,8
(i)	GO:1901360	organic cyclic compound metabolic process	5098	61	37,4	23,6	9,30E-06	0,004	1,6
(i)	GO:0008015	blood circulation	347	12	2,5	9,5	9,60E-06	0,004	4,7
(i)	GO:0003013	circulatory system process	348	12	2,6	9,4	9,90E-06	0,004	4,7
(i)	GO:0004857	enzyme inhibitor activity	291	11	2,1	8,9	1,00E-05	0,004	5,2
(i)	GO:0043491	protein kinase B signaling cascade	102	7	0,7	6,3	1,00E-05	0,004	9,4
(i)	GO:2000145	regulation of cell motility	410	13	3	10	1,00E-05	0,004	4,3
(i)	GO:0050727	regulation of inflammatory response	188	9	1,4	7,6	1,00E-05	0,004	6,5
(i)	GO:0050670	regulation of lymphocyte proliferation	143	8	1	7	1,00E-05	0,004	7,6
(i)	GO:1901215	negative regulation of neuron death	103	7	0,8	6,2	1,10E-05	0,004	9,3
(i)	GO:0032944	regulation of mononuclear cell proliferation	144	8	1,1	6,9	1,10E-05	0,004	7,6
(ii)	GO:0034976	response to endoplasmic reticulum stress	109	17	1,1	15,9	5,80E-16	<0.001	15,8
(ii)	GO:0006986	response to unfolded protein	125	15	1,2	13,8	1,70E-12	<0.001	12,1
(ii)	GO:0035966	response to topologically incorrect protein	132	15	1,3	13,7	3,80E-12	<0.001	11,5
(ii)	GO:0006984	ER-nucleus signaling pathway	94	13	0,9	12,1	8,50E-12	<0.001	14
(ii)	GO:0030968	endoplasmic reticulum unfolded protein response	83	12	0,8	11,2	3,20E-11	<0.001	14,6
(ii)	GO:0034620	cellular response to unfolded protein	84	12	0,8	11,2	3,70E-11	<0.001	14,4
(ii)	GO:0035967	cellular response to topologically incorrect protein	89	12	0,9	11,1	7,50E-11	<0.001	13,6
(ii)	GO:0033554	cellular response to stress	1155	35	11,4	23,6	2,40E-09	<0.001	3,1
(ii)	GO:0042221	response to chemical stimulus	2611	53	25,8	27,2	1,30E-07	<0.001	2,1
(ii)	GO:0006987	activation of signaling protein activity involved in unfolded protein response	62	8	0,6	7,4	1,70E-07	<0.001	13
(ii)	GO:0032075	positive regulation of nuclease activity	64	8	0,6	7,4	2,20E-07	<0.001	12,6
(ii)	GO:0032069	regulation of nuclease activity	70	8	0,7	7,3	4,40E-07	0,002	11,5
(ii)	GO:0010033	response to organic substance	1788	40	17,7	22,3	6,50E-07	0,002	2,3
(ii)	GO:0070887	cellular response to chemical stimulus	1620	37	16	21	1,20E-06	0,002	2,3
(ii)	GO:0006950	response to stress	2762	52	27,3	24,7	1,90E-06	0,002	1,9
(ii)	GO:0044723	single-organism carbohydrate metabolic process	522	18	5,2	12,8	4,60E-06	0,004	3,5
(iii)	GO:0048518	positive regulation of biological process	3286	36	15,1	20,9	1,00E-07	<0.001	2,4
(iii)	GO:0045893	positive regulation of transcription, DNA-dependent	973	18	4,5	13,5	3,40E-07	<0.001	4
(iii)	GO:0051254	positive regulation of RNA metabolic process	1018	18	4,7	13,3	6,60E-07	<0.001	3,8
(iii)	GO:0010628	positive regulation of gene expression	1054	18	4,8	13,2	1,10E-06	<0.001	3,7

(iii)	GO:0048584	positive regulation of response to stimulus	1091	18	5	13	1,80E-06	<0.001	3,6
(iii)	GO:0045944	positive regulation of transcription from RNA polymerase II promoter	684	14	3,1	10,9	2,60E-06	<0.001	4,5
(iii)	GO:0045935	positive regulation of nucleobase-containing compound metabolic process	1141	18	5,2	12,8	3,40E-06	<0.001	3,4
(iii)	GO:0010557	positive regulation of macromolecule biosynthetic process	1146	18	5,3	12,7	3,60E-06	<0.001	3,4
(iii)	GO:0051173	positive regulation of nitrogen compound metabolic process	1160	18	5,3	12,7	4,20E-06	<0.001	3,4
(iii)	GO:0048522	positive regulation of cellular process	2993	31	13,8	17,2	4,30E-06	<0.001	2,3
(iii)	GO:0080090	regulation of primary metabolic process	4014	37	18,4	18,6	5,00E-06	<0.001	2
(iii)	GO:0006357	regulation of transcription from RNA polymerase II promoter	1185	18	5,4	12,6	5,70E-06	<0.001	3,3
(iii)	GO:0042221	response to chemical stimulus	2611	28	12	16	8,10E-06	0,002	2,3
(iii)	GO:0031328	positive regulation of cellular biosynthetic process	1220	18	5,6	12,4	8,50E-06	0,002	3,2
(iii)	GO:0006355	regulation of transcription, DNA-dependent	2639	28	12,1	15,9	1,00E-05	0,004	2,3
(iii)	GO:0044707	single-multicellular organism process	5039	42	23,2	18,8	1,00E-05	0,004	1,8
(iii)	GO:0009891	positive regulation of biosynthetic process	1239	18	5,7	12,3	1,10E-05	0,004	3,2
(iii)	GO:0048583	regulation of response to stimulus	2196	25	10,1	14,9	1,10E-05	0,004	2,5
(iii)	GO:0050789	regulation of biological process	7458	54	34,3	19,7	1,10E-05	0,004	1,6
(iii)	GO:2001141	regulation of RNA biosynthetic process	2657	28	12,2	15,8	1,10E-05	0,004	2,3
(iii)	GO:0009893	positive regulation of metabolic process	1934	23	8,9	14,1	1,30E-05	0,004	2,6
(iii)	GO:0050794	regulation of cellular process	7076	52	32,5	19,5	1,40E-05	0,004	1,6
(iii)	GO:0010604	positive regulation of macromolecule metabolic process	1804	22	8,3	13,7	1,50E-05	0,004	2,7
(iii)	GO:0051252	regulation of RNA metabolic process	2712	28	12,5	15,5	1,70E-05	0,004	2,2
(iii)	GO:0031325	positive regulation of cellular metabolic process	1835	22	8,4	13,6	1,90E-05	0,004	2,6
(iii)	GO:0031323	regulation of cellular metabolic process	4071	36	18,7	17,3	2,00E-05	0,004	1,9
(iv)	GO:0009058	biosynthetic process	4754	73	45,5	27,5	4,00E-06	0,002	1,6
(v)	GO:0048585	negative regulation of response to stimulus	748	18	4,9	13,1	1,60E-06	0,004	3,7
(v)	GO:0009888	tissue development	1211	23	7,9	15,1	2,90E-06	0,004	2,9
(vi)	GO:0090304	nucleic acid metabolic process	4054	39	19,7	19,3	4,30E-06	<0.001	2
(vi)	GO:0044249	cellular biosynthetic process	4623	42	22,5	19,5	6,30E-06	<0.001	1,9
(vi)	GO:0034645	cellular macromolecule biosynthetic process	3798	37	18,5	18,5	6,70E-06	<0.001	2
(vi)	GO:0009892	negative regulation of metabolic process	1386	20	6,7	13,3	8,60E-06	0,002	3

(vi)	GO:1901576	organic substance biosynthetic process	4696	42	22,9	19,1	9,60E-06	0,002	1,8
(vi)	GO:0010605	negative regulation of macromolecule metabolic process	1294	19	6,3	12,7	1,20E-05	0,002	3
(vi)	GO:0009058	biosynthetic process	4754	42	23,1	18,9	1,30E-05	0,002	1,8
(vi)	GO:0009059	macromolecule biosynthetic process	3909	37	19	18	1,30E-05	0,002	1,9
(vi)	GO:2000113	negative regulation of cellular macromolecule biosynthetic process	868	15	4,2	10,8	1,80E-05	0,002	3,6
(vi)	GO:2000112	regulation of cellular macromolecule biosynthetic process	2876	30	14	16	2,10E-05	0,002	2,1
(vi)	GO:0005634	nucleus	5382	45	26,2	18,8	2,20E-05	0,002	1,7
(vi)	GO:0031326	regulation of cellular biosynthetic process	3065	31	14,9	16,1	2,70E-05	0,004	2,1
(vi)	GO:0051171	regulation of nitrogen compound metabolic process	3223	32	15,7	16,3	2,80E-05	0,004	2
(vi)	GO:0010558	negative regulation of macromolecule biosynthetic process	902	15	4,4	10,6	2,80E-05	0,004	3,4
(vi)	GO:0006351	transcription, DNA-dependent	2914	30	14,2	15,8	2,80E-05	0,004	2,1
(vii)	GO:0005694	chromosome	609	23	4,8	18,2	3,70E-10	<0.001	4,8
(vii)	GO:0044427	chromosomal part	517	21	4	17	6,30E-10	<0.001	5,2
(vii)	GO:0005634	nucleus	5382	76	42,1	33,9	2,30E-09	<0.001	1,8
(vii)	GO:0043229	intracellular organelle	9593	109	75	34	3,70E-09	<0.001	1,5
(vii)	GO:0043231	intracellular membrane-bounded organelle	8764	103	68,5	34,5	3,80E-09	<0.001	1,5
(vii)	GO:0043227	membrane-bounded organelle	8794	103	68,8	34,2	4,80E-09	<0.001	1,5
(vii)	GO:0051276	chromosome organization	683	22	5,3	16,7	1,70E-08	<0.001	4,1
(vii)	GO:0003676	nucleic acid binding	2962	46	23,2	22,8	1,70E-06	0,002	2
(vii)	GO:0006259	DNA metabolic process	838	21	6,6	14,4	2,30E-06	0,002	3,2
(vii)	GO:0044424	intracellular part	11026	112	86,2	25,8	3,40E-06	0,002	1,3
(vii)	GO:0005622	intracellular	11217	113	87,7	25,3	4,40E-06	0,004	1,3
(viii)	GO:0022403	cell cycle phase	752	20	3,8	16,2	7,70E-10	<0.001	5,3
(viii)	GO:0065004	protein-DNA complex assembly	136	10	0,7	9,3	1,60E-09	<0.001	14,6
(viii)	GO:0022402	cell cycle process	1022	22	5,1	16,9	4,80E-09	<0.001	4,3
(viii)	GO:0071824	protein-DNA complex subunit organization	155	10	0,8	9,2	5,70E-09	<0.001	12,8
(viii)	GO:0031497	chromatin assembly	118	9	0,6	8,4	7,90E-09	<0.001	15,2
(viii)	GO:0006333	chromatin assembly or disassembly	136	9	0,7	8,3	2,80E-08	<0.001	13,2
(viii)	GO:0006334	nucleosome assembly	110	8	0,6	7,4	8,10E-08	<0.001	14,5
(viii)	GO:0000786	nucleosome	74	7	0,4	6,6	8,90E-08	<0.001	18,8
(viii)	GO:0006323	DNA packaging	156	9	0,8	8,2	9,00E-08	<0.001	11,5
(viii)	GO:0000278	mitotic cell cycle	729	17	3,7	13,3	1,20E-07	<0.001	4,6
(viii)	GO:0000279	M phase	504	14	2,5	11,5	2,10E-07	<0.001	5,5
(viii)	GO:0000087	M phase of mitotic cell cycle	357	12	1,8	10,2	2,30E-07	<0.001	6,7
(viii)	GO:0034728	nucleosome organization	129	8	0,6	7,4	2,80E-07	<0.001	12,3
(viii)	GO:0051301	cell division	449	13	2,3	10,7	3,80E-07	<0.001	5,8
(viii)	GO:0007049	cell cycle	1314	22	6,6	15,4	4,10E-07	<0.001	3,3
(viii)	GO:0071103	DNA conformation change	189	9	1	8	4,60E-07	<0.001	9,5
(viii)	GO:0007067	mitosis	346	11	1,7	9,3	1,30E-06	<0.001	6,3

(viii)	GO:0000280	nuclear division	346	11	1,7	9,3	1,30E-06	<0.001	6,3
(viii)	GO:0032993	protein-DNA complex	115	7	0,6	6,4	1,80E-06	<0.001	12,1
(viii)	GO:0044427	chromosomal part	517	13	2,6	10,4	1,80E-06	<0.001	5
(viii)	GO:0034622	cellular macromolecular complex assembly	526	13	2,6	10,4	2,20E-06	<0.001	4,9
(viii)	GO:0048285	organelle fission	369	11	1,9	9,1	2,40E-06	<0.001	5,9
(viii)	GO:0005694	chromosome	609	13	3,1	9,9	1,10E-05	0,002	4,2

	cell death
	signaling/response to stimulus
	cell proliferation/motility/differentiation
	biosynthetic/metabolism processes
	nuclear/ organelle/ chromatin modifications
	cell cycle
