# Supplementary material for: Senescence-secreted factors activate Myc and sensitize pretransformed cells to TRAIL-induced apoptosis
Source: Aging Cell. 2014 Mar 4;13(3):487–96. doi: 10.1111/acel.12197 (PMC4326894; doi:10.1111/acel.12197)
Supplement: Supplementary file 9 — Table S2 Primers used in quantitative PCR. [file acel0013-0487-sd9.pdf]

Table S2. Primers used in Quantitative PCR

$FLIP_L$ forward	5'-TGCCTCAGAGCATACCTGAAGAGA-3'
$FLIP_L$ reverse	5'-TCTCGAAGAAGCTCTGTCTCATTGC-3'
GAPDH forward	5'-ATGACATCAAGAAGGTGGTGA-3'
GAPDH reverse	5'-CTGTAGCCAAATTCGTTGTCA-3'
IL6 receptor forward	5'-GGGCTGGAACGGTCAAAGACA-3'
IL6 receptor reverse	5'-GGATGACACAGTGATGCTGGA-3'
36B4 forward	5'-GATTGGCTACCCAACTGTTG-3'
36B4 reverse	5'-CAGGGGCAGCAGCCACAAA-3'
IL6 forward	5'-CCAGGAGCCCAGCTATGAAC-3'
IL6 reverse	5'-CCCAGGGAGAAGGCAACTG-3'
IL8 forward	5'-TTGGCAGCCTTCCTGATTTC-3'
IL8 reverse	5'-TCTTTAGCACTCCTTGGCAAAAC-3'
