# Supplementary material for: Senescence-secreted factors activate Myc and sensitize pretransformed cells to TRAIL-induced apoptosis
Source: Aging Cell. 2014 Mar 4;13(3):487–96. doi: 10.1111/acel.12197 (PMC4326894; doi:10.1111/acel.12197)
Supplement: Supplementary file 10 — Data S1 Experimental procedures. [file acel0013-0487-sd10.pdf]

Supplementary Experimental Procedures

Cell culture and reagents. BJ system cells were cultured in DMEM 1g/L glucose/ Medium 199 (4:1) containing 15% of fetal calf serum and gentamicin. HEK system cells were cultured in DMEM 1g/L glucose, containing 10% of fetal calf serum and gentamicin. WI-38, HEK 293, T47D and HepG2 cells were obtained from the in-house cell culture service. WI-38 cells were grown in MEM 15% of fetal calf serum with gentamicin and 2x (Non-essential amino acids and vitamins - Invitrogen). HEK293 cells were grown in DMEM 1g/L glucose containing 10% of fetal calf serum and gentamicin + Penicillin-Streptomycin. HepG2 cells were grown in Eagle 10% of fetal calf serum with, Non-essential amino acids, 1mM Sodium Pyruvate, 2 mM L-Glutamine and gentamicin.

Generation of senescent cells. To generate senescent (T47D-S) cells, T47D cells were treated with 0.2 μM doxorubicin for 24h, after which media was changed and cells were grown without the chemical for the next 10 days after which growth arrested, senescent cells were obtained. Both T47D and T47D-S cells were grown in RPMI 1640 medium without HEPES containing 10% of fetal calf serum, insulin (0.6 μg/ml) and gentamicin.

H_2O_2- and Etoposide-induced senescencent cells were obtained in similar ways. WI-38 cells were treated with Etoposide (20μM for 24h) or with H_2O_2 (150 μM for 2h) after which medium was regularly changed and WI-38 cells were let to grow for 10 days before senescent cells were obtained.

Retroviral production and infection. Retroviruses were produced in HEK 293 cells with *pCLII Ampho* helper plasmid and respective retroviral plasmid, using the Fugene6 Transfection Reagent (Roche). After 48 hours, the medium containing viral particles was collected and filtered through a 0.4 μm filter and used in the presence of 8μg/ml polybrene. 48 h after transduction,viral supernatant was replaced with fresh medium and selection with puromycin (1μg/ml) was initiated to obtain successfully transduced polyclonal population.

Cell and tissue staining. β-Gal staining was performed using Senescence β-Galactosidase Staining Kit #9860 (Cell Signaling) and senescent cells were visualized by perinuclear blue staining. Senescence associated Heterochromatic Foci (SAHFs) were visualized by DAPI, as described (Narita *et al.* 2003).

Immunofluorescence and immunohistochemistry. Cells were fixed with 1% PFA, permeabilised using (0.25% Triton X100 for 10 min at room temperature) and blocking was performed in 5% BSA for 30 min at room temperature. Cells were incubated with primary antibody in a humidified

chamber overnight at 4 °C and then secondary antibody (1/500) in 1% BSA at room temperature for 1h.

For immunohistochemistry, in brief, tumor tissue sections were first de-paraffinized using a Histosol protocol (Kedinger *et al.* 2011) and rehydrated through gradients of ethanol and distilled water. Antigen retrieval was performed in 10mM sodium citrate at pH 6 by boiling the slides in glass chambers. Incubation with antibodies was performed as previously described.

Transient transfections. Cells were seeded either in 24 (80,000 cells/well) or in 6 well plates (400,000 cells/well). Transfections were performed with either 0.8µg/well (24 well plates) or 4µg/well (6 well plates) of appropriate plasmid and using 2µl (24 well plates) or 10 µl (6 well plates) of Lipofectamine 2000 (Invitrogen). Plasmids used were as follows: pBabe puro and pBabe Ras puro G12V (kind gifts from Dr. *Weinberg*), *pGFPmax* (Amaxa), pBabe Myc ER, pCMV-ß-galactosidase, pGL3-FLIP promoter-luciferase reporter plasmid (Addgene, plasmid 16016), pGL3-basic.

Luciferase reporter assays. Cells were seeded in 6 well plates (4 x 10^5cells/well) and transfected by using Lipofectamine 2000 (Invitrogen) according to manufacturer's protocol and total amount of 4 µg of DNA. Three different plasmids were used at the same time: first, pCMV-ß-galactosidase plasmid to measure transfection efficiency (5% of total plasmid amount; 0.2 µg), second pGL3-basic / pGL3-FLIP promoter-luciferase reporter plasmid (Addgene plasmid 16016) (45% of total plasmid amount; 1.8 µg) and third MycER / pBabe (50% of total plasmid amount; 2 µg). After transfection cells were reseeded into 24 well plates, (50,000 cells/well) and were grown with 4-hydroxytamoxifen (4-OHT) at final concentration of 10^{-6} M. After attaching, cells were incubated with conditioned medium from senescent cells as mentioned, with each sample being present in triplicate. Luciferase activity was measured using the Luciferase assay system (Promega) and light units were normalized to ß-galactosidase activity to control transfection efficiency.

Western blotting and antibodies. Whole cell protein extract was prepared using lysis buffer comprising 0.5M LSBD (0.5 M NaCl, 50mM Tris-HCl pH 7.9, 20% glycerol, 1% NP-40, 1mM DTT), 0.3% NP-40, 1 X Protease Inhibitor Cocktail (Roche), 1mM NaF, 10 mM Na_3VO_4, 1mM PMSF, 0.125 µM of Okadaic Acid. Protein concentration of extracts was measured using a Protein Assay reagent (Bio-Rad Laboratories). Fifty µg proteins were separated by SDS PAGE, transferred to nitrocellulose membranes and incubated with indicated antibodies. Western blots were quantified using Image J and normalized to a loading control. Antibodies used were as follows: FLIP (NF6; Mouse IgG1; Alexis; 804-428), c-Myc (N-262; Rabbit; Santa Cruz; sc-764) and (D84C12 XP; Rabbit, Cell Signaling #5605), caspase-8 (1C12; Mouse; Cell Signaling; #9746), caspase-9 (Rabbit,

Cell Signaling; #9502), PARP (Rabbit, Cell Signaling; #9542), HRas (Rabbit, sc-520, Santa Cruz), β-actin (C-11; Goat; Santa Cruz; sc-1615), SV40 T (Pab 108; Mouse; Santa Cruz, sc-148), Phospho-Stat3 Tyr705 (Rabbit, Cell Signaling, #9131), Stat3 (Rabbit, Cell Signaling, #9132). The IL6 neutralizing antibody (R&D systems, AB-206-NA) was used at final concentration of 10μg/ml.

Properties of TRAIL-sensitizing senescent factor. Several rounds of freeze/thaw or boiling the CMS (100°C, 10 min) did not affect the activity of CMS on pre-transformed cells of both stepwise systems. Ultracentrifugation of total conditioned media from senescent cells was performed using Amicon filter tubes (Ultra-15, Millipore, Billerica, MA, USA) with a 3kDa cutoff, (5000 rcf, for 2h). After ultracentrifugation total CMS and its lower 3kDa fraction were incubated with cells as usual. For protein degradation assays mix of several enzymes (Trypsin/EDTA 0.25%, Sigma-Aldrich, final 0.05%; Carboxypeptidase Y, C-3888, Sigma-Aldrich and α-Chymotrypsin, C-4129, Sigma-Aldrich) each used at final enzyme/peptide ratio = 1/100, was incubated with either TRAIL-mimicking "m1d" peptide (Pavet *et al.* 2010) (generous gift from Prof. Gilles Guichard, IECB, Bordeaux, France), or the lower 3kDa fraction of CMS for 3h at room temperature.

Xenograft experiments. We injected equivalent numbers of either transformed (HA1ER) or pre-transformed (HA1E) cells into nude mice and waited for tumor development for three weeks. In parallel, equivalent numbers (2 x 10^6) of pre-transformed and either young replicating or senescent cells were pre-mixed and injected into nude mice. Tumors were left to grow for approximately three weeks prior to excision and immunostaining.

References

Kedinger V, Muller S, Gronemeyer H (2011). Targeted Expression of Tumor Necrosis Factor-Related Apoptosis-Inducing Ligand TRAIL in Skin Protects Mice Against Chemical Carcinogenesis. *Mol Cancer*. **10**, 34.

Narita M, Nunez S, Heard E, Lin AW, Hearn SA, Spector DL, Hannon GJ, Lowe SW (2003). Rb-mediated heterochromatin formation and silencing of E2F target genes during cellular senescence. *Cell*. **113**, 703-716.

Pavet V, Beyrath J, Pardin C, Morizot A, Lechner MC, Briand JP, Wendland M, Maison W, Fournel S, Micheau O, Guichard G, Gronemeyer H (2010). Multivalent DR5 peptides activate the TRAIL death pathway and exert tumoricidal activity. *Cancer Res*. **70**, 1101-1110.
